# Impact of Ecological Restoration on the Physicochemical Properties and Bacterial Communities in Alpine Mining Area Soils

**DOI:** 10.3390/microorganisms12010041

**Published:** 2023-12-25

**Authors:** Lingjian Kong, Lin Zhang, Yingnan Wang, Zhanbin Huang

**Affiliations:** School of Chemical and Environmental Engineering, China University of Mining and Technology (Beijing), Beijing 100083, China; ljkong2023@163.com (L.K.); wendyl0902@163.com (L.Z.); wangyingsouth@163.com (Y.W.)

**Keywords:** spatial sequence, high-throughput sequencing, 16S rRNA amplicon study, soil organic, proteobacteria, bacillus, early-stage enhancements

## Abstract

Ecological restoration has notably impacted microbe and soil characteristics in abandoned open pit mines, especially in alpine regions. Yet, the adaptive responses of microbial communities in the initial years of mine site restoration remain largely unexplored. This study endeavors to offer a thorough comprehension of soil properties and microbial dynamics during the initial phases of alpine mining land reclamation. It places emphasis on physicochemical properties and microbial community composition and evaluates the feasibility of phytoremediation, along with proposing subsequent measures. Our study employs spatial sequence instead of time-sequenceal sequence to investigate early-stage changes in soil microbes and physicochemical properties in alpine mining land reclamation. We used high-throughput sequencing for the 16S rRNA amplicon study. Over time, soil physicochemical properties improved noticeably. Soil pH shifted from neutral to alkaline (7.04–8.0), while soil electrical conductivity (EC) decreased to 77 μS·cm^−1^ in R_6a. Cation exchange capacity (CEC) initially decreased from R_2a (12.30–27.98 cmol·kg^−1^) and then increased. Soil organic matter increased from 17.7 to 43.2 g·kg^−1^ over time during mine reclamation and restoration. The dominant bacterial community consisted of *Proteobacteria* (33.94% to 52.09%), *Acidobacteriota* (4.94% to 15.88%), *Bacteroidota* (6.52% to 11.15%), *Actinobacteriota* (7.18% to 9.61%), and *Firmicutes* (4.52% to 16.80%) with varying relative abundances. Gene annotation of sequences from various reclamation years revealed general function prediction, translation, ribosome structure, cell wall/membrane/envelope biogenesis, nucleotide translocation, and metabolism, along with other related functions. Mine reclamation improved soil fertility and properties, with the R_6a treatment being the most effective. Starting in the 2nd year of reclamation, the effective phosphorus content and the dominance of microbial bacteria, notably the *Bacillus* content, decreased. Firmicute fertilization promoted phosphorus and bacterial growth. In conclusion, employing a blend of sequencing and experimental approaches, our study unveils early-stage enhancements in soil microbial and physicochemical properties during the reclamation of alpine mining areas. The results underscore the beneficial impacts of vegetation restoration on key properties, including soil fertility, pore structure, and bacterial community composition. Special attention is given to assessing the effectiveness of the R_6a treatment and identifying deficiencies in the R_2a treatment. It serves as a reference for addressing the challenges associated with soil fertility and microbial community structure restoration in high-altitude mining areas in Qinghai–Tibet. This holds great significance for soil and water conservation as well as vegetation restoration in alpine mining regions. Furthermore, it supports the sustainable restoration of local ecosystems.

## 1. Introduction

Agriculture and mining are ancient human endeavors vital to modern civilization [1]. Open-pit mining yields valuable metals but also harms the environment, affecting both surface and subsurface ecosystems [2]. Research typically focuses on soil improvement for grass planting in lower altitudes rather than in alpine mining regions. Given the challenges posed by high altitudes, limited soil resources, and costly soil transportation in these areas, a practical approach for revegetation involves using local residual soils, amending them, and directly planting grasses [3]. This technology can help reduce mining’s toxic impact on ecosystems [4]. Appropriate measures such as fertilization increase the population of the corresponding microorganisms, and thus the soil properties show the potential for ecological restoration of mining areas. Soil fertilization enhances bioremediation of contaminated mine soils by stimulating microbial activity, improving nutrient availability, fostering plant-microbe interactions, increasing rhizosphere activity, boosting soil enzyme function, improving soil structure, stimulating indigenous microbial populations, and accelerating natural attenuation processes.

Soils are vital for ecosystem maintenance, offering a foundation for their health and stability through diverse prokaryotic communities [5]. Root-associated microbes are pivotal for crop performance [6,7,8]. These inter-root communities mediate plant-soil interactions, essential for nutrient cycling, disease resistance, and substrate metabolism [9,10,11]. By conducting an in-depth study on the interplay between plants and soil microorganisms, coupled with soil enhancement, ecosystem engineering, and comprehensive data analysis, we can expedite the restoration of degraded areas, enhance soil quality, and stabilize ecosystems. Analyzing the spatial-temporal dynamics of soil microorganisms on reclaimed mining land and their influencing factors offers insights into soil quality improvement [12]. Reclamation timing impacts soil microbes and plant health. The utilization of early indicators, such as soil bulk density, porosity, field water holding capacity, pH, electrical conductivity, nutrients, and microbial populations, can aid in forecasting changes in soil quality during mine reclamation. In Western Gujarat, India, a chronosequence-based phytoremediation-assisted restoration approach was implemented to comprehend microbial community succession and functioning changes. This time-series phytoremediation method monitors temporal and age-related shifts in soil properties, microbial diversity, and communities as plants grow while maintaining constant ecological conditions. Utilizing resident microorganisms linked to tailings soils and reclaimed land as a prospective tool reveals insights into the spatial distribution of microbial communities [13]. Through ecological protection measures like flower planting, turf slope preparation, reforestation, and mine park greening, combined with phylogenetic investigations using PICRUSt with 16S rRNA sequences to predict soil bacterial community function, Zijin Mine has successfully achieved ecological restoration. This approach effectively mitigated and restored the ecological and environmental impacts in the mining area [14]. Yet, mechanisms sustaining soil microbes in high-alpine mining areas need further study [15]. 

Bacteria are diverse and abundant in soil, playing crucial roles in organic matter decomposition, nutrient cycling, nitrogen fixation, and plant support, thus maintaining ecosystem functions [16,17]. Grass species affect soil bacterial communities by changing environmental conditions and nutrient availability, influencing soil ecosystem functionality and resilience [18,19]. Historically, research on soil microbial populations in alpine mining areas has been limited, resulting in an incomplete understanding of ecological restoration. Our goal is to assess changes in the physical and chemical properties of soils in alpine mining areas, considering factors such as remediation strategies. By analyzing changes in soil structural properties and bacterial communities and identifying key factors influencing these changes, we aim to gain in-depth insights into the mechanisms of dynamic soil changes in alpine mining ecosystems. The main objectives of this study are: (1) Assess soil physicochemical changes. (2) Examine shifts in soil bacterial communities. (3) Identify the factors influencing these changes. We aim to enhance understanding, provide a scientific basis for ecological restoration in alpine mining regions, and predict bacterial community evolution in response to restoration duration. We hypothesize that functional soil responses will align with bacterial community shifts.

## 2. Materials and Methods

### 2.1. Description of the Research Center

The Qinghai West Copper Mine, located in China’s Qinghai Province in the Delni Mountains of Dawu Township, Maqin County, is situated on a high-altitude plateau. The region has varying elevations, with an average height of 4200 m and a reduced air pressure (0.6 atmospheres). It experiences a highland continental climate with cold and warm seasons. The cold season lasts from October to April, with temperatures dropping to −26.3 °C and a relative humidity of 40–48%. The warm season spans from May to September, with an average temperature of 5.5 °C, peaking at 19.7 °C, and showing a daily temperature fluctuation of 25.1 °C. Rainfall mainly occurs during the warm season, contributing to an average annual precipitation of 375.2 mm, with over 80% being heavy rainfall. The area is predominantly covered by alpine meadow-type flora, including *Elymus nutans*, *Poa crymophila*, *Elymus sibiricus*, *and Festuca sinensis* [20]. These natural grasses are crucial pastureland for local herders, supporting their livelihoods. A description of the nature of the soils in the study area can be found in the attached Text S1. Additional information, such as cropping systems and geographical details for each year, is provided in Table 1.

### 2.2. Sample Collection

**Figure 1:**
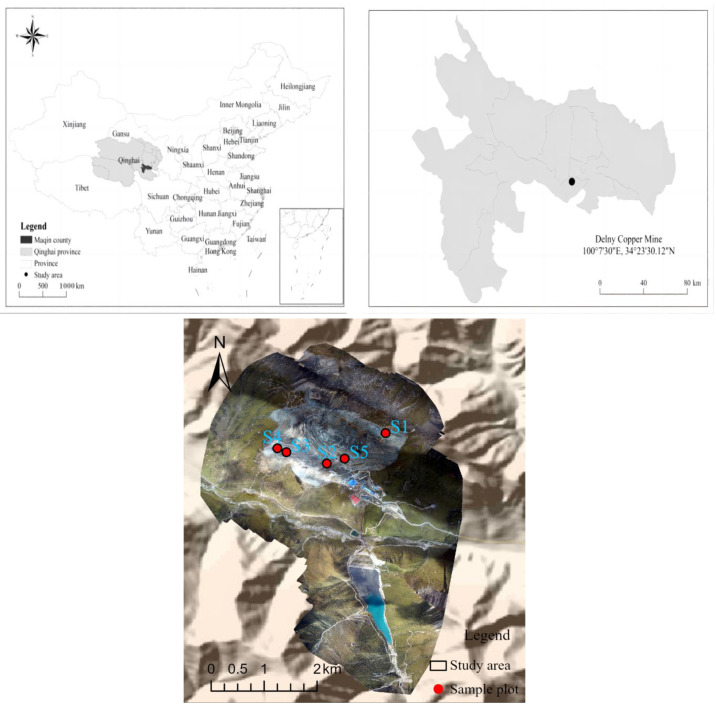
Study Area Location and Sampling Point Layout.

In July 2022, we employed a spatial sequence instead of a time-sequenceal sequence for sample collection during field surveys in the Qinghai West Copper Mining Area. Notably, the reclamation site was undisturbed after sampling. Surface soil samples (0–20 cm) were collected from all test sites, while the mine site’s natural turf soil (0–20 cm) served as the control (OL). Each sample plot, measuring 25 × 25 m, was sampled in triplicate, with three designated plots at each site for soil sampling. In separate 1 m × 1 m squares, three soil samples were collected and combined into one replicate, resulting in five replicates at various sampling points. These samples were transported to the laboratory for soil physical and chemical analysis, as well as microbial assessment. Soils from different reclamation years and natural turf were sieved through a 1 cm sieve to remove plant and large rock residues and stored as sub-samples. A portion of the soil was further sieved to 2 mm for physicochemical parameter analysis, including pH, Electrical Conductivity(EC), total salt content (TSC), and heavy metals. The remaining sub-samples were dried for Available Potassium (AK), Hydrolyzable Nitrogen (HN), Total Nitrogen (TN), Available Phosphorus (AP), and Soil Organic Matter (SOM) measurements. Soil samples intended for DNA extraction and sequencing were stored separately at −20 °C. The analyzed samples (R_1a, R_2a, R_4a, R_6a, and OL, as detailed in Table 1) correspond to S1, S2, S3, S4, and S5 in Figure 1.

### 2.3. Determination of Soil Physical and Chemical Properties

We conducted a comprehensive analysis of the physical and chemical compositions, as well as the major heavy metal contents, of substrates from different reclamation years within the mining area. The physical parameters examined included soil bulk density (SBD), soil specific gravity (SPG), total soil porosity (TSP), soil capillary porosity (SCP), pH, EC, dry matter (DM), cation exchange capacity (CEC), total salt content, and nutrient content (AK, HN, TN, AP, and SOM). Additionally, we determined the chemical characteristics, specifically the heavy metal content, which encompassed elements such as As, Hg, Cd, Cr, and Pb in the soil substrates. 

SBD, TSP, soil field water holding capacity (SWC), and other physical parameters were determined using the ring knife method. pH was measured in a 1:2.5 soil/water suspension using a combined pH electrode, and EC was assessed in a 1:5 soil/water suspension employing a conductivity meter. The total soil salt content was determined in 1:5 soil/water suspensions through the residue drying-mass method. Soil cation exchange was assessed by leaching the soil with a hexamine cobalt trichloride solution as the leaching agent, maintained at (20 ± 2) °C [21]. We analyzed SOM, DM, HN, TN, AP, and AK content using established techniques for rock mineral analysis. TN levels were determined using an elemental analyzer, while DM content was assessed via the drying method. SOM content was determined through the K_2_Cr_2_O_7_/H_2_SO_4_ wet digestion method, with an organic carbon-to-organic matter conversion coefficient of 1.724. HN content was measured using the Kjeldahl nitrogen tester method, while AP levels were assessed according to HJ704-2014, employing the sodium bicarbonate leaching-molybdenum and antimony anti-spotting spectrophotometric method. AK was determined by flame atomic absorption spectrometry. To evaluate the total contents of Cu, Zn, Pb, Ni, and Cr in the soil, flame atomic absorption spectrometry was employed. Hg and As were determined through microwave digestion/atomic fluorescence, and Cd levels were assessed using graphite furnace atomic absorption spectrophotometry.

Before assessing physical properties, soil samples were air-dried at room temperature and sieved through a 2 mm mesh. The outcomes of the experiment were derived using the ring knife method, as previously described. Specific results were calculated using the following formula:

Soil bulk density = dry soil weight in the ring knife (g)/ring knife volume (cm^3^) (1)

Soil capillary porosity = {(water absorption for about 2 h with soil ring knife weight (g) − ring knife weight (g) − dry soil weight in the ring knife (g)/ring knife volume (cm^3^)} × 100% (2)

(3) $$\rho_{s}=\frac{m_{s}}{m_{s}+m_{bwl}-m_{bwsl}}\rho_{wl}$$

where: ρ_s_ represents the soil specific gravity, g·cm^−3^; ρ_wl_ represents the density of distilled water at t degrees, g·cm^−3^; m_s_ represents the mass of dried soil sample, g; m_bw1_ represents the mass of specific gravity bottle + water at t degrees, g; m_bws1_ represents the mass of specific gravity bottle + water + soil sample at t degrees, g.

Field water holding capacity = {(water absorption for about 2 h with soil ring knife weight (g) − ring knife weight (g) − dry soil weight in the ring knife (g)/dry soil weight in the ring knife (g))} × 100%(4)

Total soil porosity = {(ring knife weight with soil around 6 h of immersion (g) − ring knife weight (g) − dry soil weight inside ring knife (g)/ring knife volume (cm^3^)} × 100% (5)

Cation exchange capacity (*CEC*) calculation formula:

(6) $$CEC=\frac{(A_{0}\;-\;A)\;\times \;V\;\times \;3}{b\;\times \;m\;\times \;W_{\mathrm{dm}}}$$

where: *A*_0_ represents the absorbance of the blank sample; *A* represents the absorbance of the sample or corrected absorbance; *V* represents the volume of leachate, mL; 3 refers to the charge number of [Co(NH_3_)_6_]^3+^; b represents the slope of the standard curve; m represents the amount of sample, g; and W_dm_ represents the content of dry matter in the soil sample, %.

### 2.4. DNA Extraction and PCR Amplification

Genomic DNA from alpine mine soil microbial communities was extracted using the E.Z.N.A.^®^ soil DNA kit (Omega Bio-tek, Norcross, GA, USA). DNA quality and concentration were assessed through agarose gel electrophoresis and a NanoDrop 2000 UV-Vis spectrophotometer (Thermo Scientific, Wilmington, DC, USA). For bacterial community analysis, the extracted DNA was used as a template for PCR amplification of the full-length 16S rRNA gene. The primers 27F [22] and 1492R (5’-RGYTACCTTGTTACGACTT-3’) [23] were employed for full-length 16S rRNA gene amplification, following the PCR reaction steps attached in Text S2.

### 2.5. DNA Library Construction and Sequencing

The library construction was performed using the SMRTbell^®^ Express Template Prep Kit 2.0, involving the following steps: (1) Addition of the Illumina Official Junction Sequence to the target region via PCR; (2) Recovery of the PCR product by gel excision using the Gel Recovery Kit; (3) Elution in Tris-HCl buffer and agarose gel electrophoresis for detection; (4) Denaturation in sodium hydroxide to generate single-stranded DNA fragments. Sequencing was conducted on the Pacbio Sequel II System (Shanghai Meiji Biomedical Technology Co., Ltd., Shanghai, China). Illumina sequencing involves a series of steps attached in Text S3.

### 2.6. Data Processing and Statistical Analysis

The data were analyzed using Excel 2021 and SPSS 26.0 for statistical analysis and graph creation (Origin 2023). We employed a one-way ANOVA with the least significant difference method to assess soil property variations across sites. Alpha diversity indices were calculated using Mothur software (version v.1.30.2) [24]. R programming was employed to generate dilution graphs, Venn diagrams, and various community analysis diagrams (bar, pie, and heatmap). We used Circos-0.67-7 for visualizing sample-species correspondence. Microbial community structure similarity between samples was assessed using PCoA using the Bray-Curtis distance algorithm. ANOSIM analysis was used to compare species abundance between samples [25]. RDA explored the relationship between soil-enriched phyla and soil properties. Evolutionary trees were constructed using FastTree (version 2.1.3) and analyzed with FastUniFrac for inter-sample distance [26]. For 16S function prediction, PICRUSt (version 2.2.0) software was used. The OTU table was imported into the Galaxy Web platform, Picrust, for predictions based on the KEGG database.

## 3. Results

### 3.1. Variations in Soil Physicochemical Properties across Reclamation Years

Descriptive statistical analysis was performed on soil physical and chemical property data from various reclamation years, indicating significant improvements in soil properties compared to the original natural turf soil. Table 2 provides a summary of the physicochemical properties of soil samples collected over different years. The soil pH was weakly alkaline across all reclamation years, higher than in the unmined natural grassland (OL). While there were variations in pH, no significant differences were observed among treatments in years R_6a, R_4a, and R_1a. In contrast, R_2a and OL had significantly lower pH levels compared to the other three treatments (*p* < 0.05). The highest pH of 8.00 was recorded in the R_4a treatment, and the lowest pH of 7.04 was recorded in the OL treatment. With increasing restoration years, soil EC initially increased and then significantly decreased (*p* < 0.05). The R_2a treatment had the highest soil EC, surpassing the OL treatment by 118.74% (*p* < 0.05). Conversely, the R_6a treatment had the lowest soil EC, 38.15% lower than OL. CEC showed a fluctuating pattern, initially decreasing and then increasing. The highest CEC was in the R_6a treatment, exceeding OL by 67.01%. In contrast, the R_2a treatment had the lowest CEC, 26.57% lower than OL.

SBD values increased over time in the order R_1a > R_6a > R_4a > R_2a > OL, with all restored soils exhibiting higher SBD than OL. STP and SWC initially increased and then decreased as restoration progressed. The R_2a treatment showed the highest values (*p* < 0.05), while R_1a remained stable, consistently lower than OL across treatments. TN values did not significantly differ among treatments (*p* > 0.05). R_1a had the highest TN, while R_2a had the lowest, 16.39% lower than OL. DM content in the treatment groups was not significantly different from OL (*p* > 0.05) and ranged from 0 to 0.8%. SOM content gradually increased with treatment duration but remained lower than OL, with R_6a being 4.16% lower than OL. HN values varied minimally (*p* < 0.05) and were lower than OL in R_1a, R_2a, and R_4a, with R_6a significantly higher (*p* < 0.05) than R_4a, though still 28.40% lower than OL. Note that OL has high organic matter. AP and AK trends were consistent across treatments, with values surpassing OL in all groups. R_1a had the highest values (*p* < 0.05), while R_4a had the lowest AP values. As reclamation duration increased, AP and AK values improved in all treatment groups, highlighting reclamation’s positive impact.

### 3.2. Changes in Heavy Metal Content of Soils with Different Years of Reclamation

Copper concentrations remained below screening values in reclaimed land, with R_1a being 28.2% lower than OL (Table 3). Hg and Cu increased with reclamation duration (*p* < 0.05), notably peaking in R_2a with concentrations 123.08% and 760% higher than OL, respectively. Some heavy metals (Cr, Ni, and Cd) showed significant increases (*p* < 0.05) in R_4a, with levels 75.90%, 251.64%, and 75% higher than OL. Pb and As exhibited irregular trends, with R_2a having the highest concentrations, surpassing OL by 50% and 125.35%, respectively, and R_4a showing the lowest values, 12.50% and 12.68% lower than OL for Pb and As, respectively.

### 3.3. Soil Bacterial Community Composition in Different Reclamation Years

Data underwent processing via the USEARCH11-uparse algorithm, resulting in 75,520 OTUs and 112,393,492 bases across the five samples. The average sequence length was 1488.33941 bp, with varying OTUs per sample (5709 to 7588) and an average of 11,225 high-quality sequences per sample. The dilution curve reached a saturation plateau at a genetic distance of 8.56, indicating adequate sequencing data; further reads will not substantially increase the OTU count.

**Figure 2:**
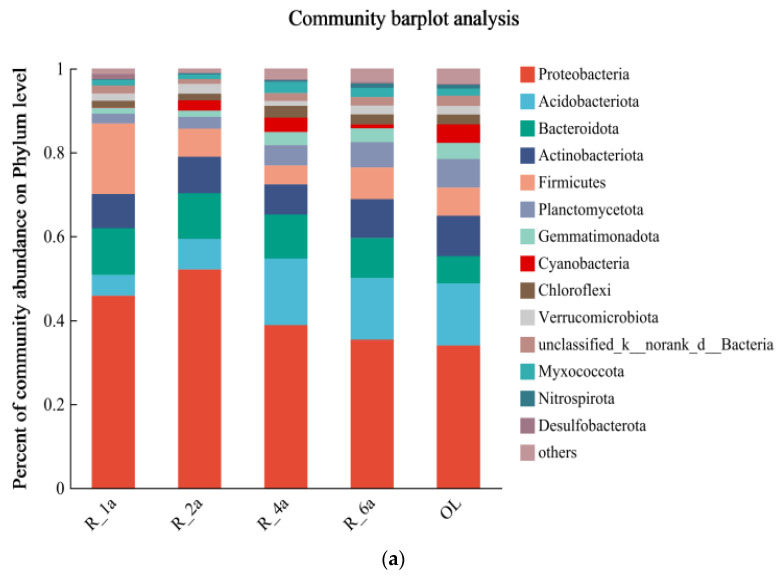

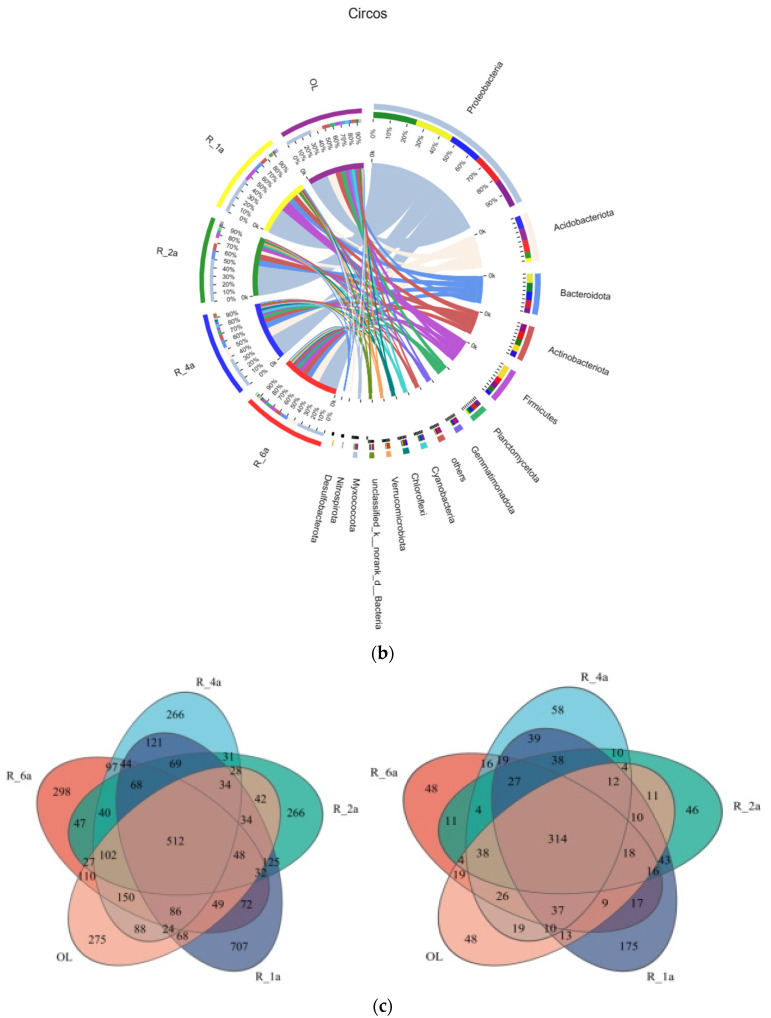
(**a**) The relative abundance of soil bacterial communities in the phylum. Note: The horizontal axis designates the sample names; the vertical axis quantifies the species proportion within each sample; distinctively colored bars symbolize different species; and the length of each bar corresponds to the proportionate size of the respective species. (**b**) The Circos Sample-Species Relationship Chart is commonly used to illustrate the distribution of microbial species in various samples. Circos plots depict samples and their subgroups on one side and the main dominant species on the other, indicating abundance distribution through inner colored bands; (**c**) Venn diagrams show unique and shared species and Genus under different treatments. Note: Unique colors signify different subgroups (or samples); overlapping numbers indicate the species count common to multiple subgroups; and non-overlapping numbers denote the number of species exclusive to the respective subgroup.

In this study, “abundant” OTUs were those with a relative abundance above 1% at the bacterial phylum and genus level. Fourteen bacterial phyla were detected across all samples. Variations in dominant bacterial phyla composition were observed when comparing different years (Figure 2a). The dominant phyla at the phylum level included *Proteobacteria* (41.24%), *Acidobacteriota* (11.53%), *Bacteroidota* (9.73%), *Actinobacteriota* (8.57%), *Firmicutes* (8.48%), and others such as *Planctomycetota*, *Gemmatimonadota*, *Cyanobacteria*, *Chloroflexi*, *Verrucomicrobiota*, *unclassified-k-norank-d-Bacteria*, *Myxococcota*, *Nitrospirota*, *and Desulfobacterota.* Significant differences (*p* < 0.05) in the relative abundance of these dominant phyla were observed across various reclamation times, with Proteobacteria consistently dominating the restored soils, although the proportions varied in each treatment (Figure 2b).

Venn diagrams illustrated species and genus distribution across treatments, demonstrating that 512 species and 314 genera were shared among all samples (Figure 2c). Furthermore, each of R_1a, R_2a, R_4a, R_6a, and OL had a specific species and genus. Specifically, R_1a, R_2a, R_4a, R_6a, and OL had 707 and 175, 266 and 46, 266 and 58, 298 and 48, and 275 and 48 specific species and genera, respectively. The analysis indicated significant differences (*p* < 0.05) in bacterial communities among soils with varying reclamation times (R_1a, R_2a, R_4a, R_6a, and OL) at the genus level and higher taxonomic levels (Figure S1).

Certain bacterial taxa were more abundant in restored soils, while others predominated in natural, unamended soils (OL) (Figure 2a). Among the enriched OTUs (representing > 1% of the sequences), 22 genera accounted for 40.4% of the total, with 11 species of enriched OTUs comprising 16.85% (Figure S3). Notably, *unclassified_g._Brevundimonas* species were exclusive to restored soils (R_1a and R_6a), while the genus *Limnobacter* and *Luteimonas_sp._R-37032* were specific to R_2a soils. Additionally, *WCHB1-32* was associated with R_1a soil, Luteimonas, and Cellvibrio with R_4a soil (Figure S1). Approximately 59.60% and 83.15% of bacterial sequencing reads could not be assigned to a known genus or species, indicating potential unique and novel lineages that warrant further phylogenetic investigation.

**Figure 3:**
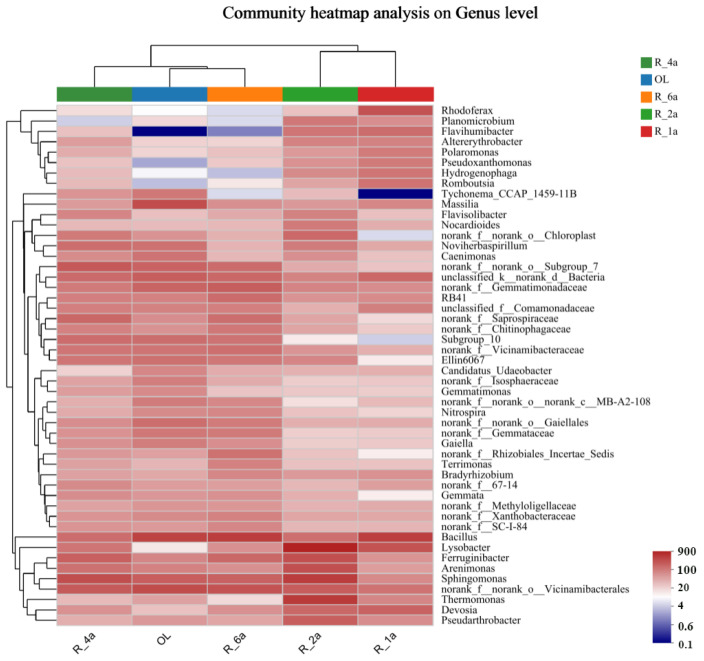
Soil bacterial interactions at the level of different land species. Note: Sample names (or subgroup names) are on the horizontal axis, species names on the vertical axis, and the color gradient of the blocks represents changes in species abundance across samples. The corresponding values for the color gradient are displayed on the right side of the figure.

In the initial treatment years, R_1a and R_2a, all seven phyla were predominant, with varying abundances. At R_1a, they included *Proteobacteria* (45.84%), *Firmicutes* (16.80%), *Bacteroidota* (11.15%), *Actinobacteriota* (8.16%), and *Acidobacteriota* (4.94%). After one year, *Proteobacteria*, *Acidobacteriota*, and *Actinobacteriota* increased by 6.25%, 2.35%, and 0.49%, respectively, while *Firmicutes* and *Bacteroidota* decreased by 10.09% and 0.24%, respectively. With continuous recovery until the natural state, R_4a, R_6a, and OL, the proportion of unclassified read segments rose from 2.63% to 3.65% (Figure 2b). The abundance of *Proteobacteria*, *Acidobacteriota*, and *Bacteroidota* gradually decreased. R_4a exhibited a 4.87%, 1.09%, and 3.96% increase compared to OL, respectively. Meanwhile, *Actinobacteriota*, *Firmicutes*, and *Planctomycetota* gradually increased, with R_4a being 2.43%, 2.23%, and 2.00% lower than OL, respectively. *Proteobacteria* predominated with a surprising 52.09% of readings (R_2a), and a minimal 0.94% remained unclassified in the hierarchy (Figure 2b). At the genus level, five dominant genera were found: *Bacillus*, *Lysobacter*, *Sphingomonas*, *norank_f__norank_o___Vicinamibacterales*, and *Ferruginibacter*. Notably, changes in the genus-level bacterial community structure were influenced by reclamation treatments. Approximately 46.64% (ranging from 36.78% to 50.52%) of the total sequencing reads remained unclassified at the genus level. Specifically, Bacillus declined from 4.65% in R_1a to 1.68% in R_2a before rebounding to 4.24% in OL (Figure S1). Lysobacter exhibited an increase from 3.10% in R_1a to 8.30% in R_2a, then dropped to 0.14% in OL. *Sphingomonas* increased from 0.97% in R_1a to 4.83% in R_2a and remained at 2.46% in OL. *Norank__f__norank__o___Vicinamibacterales* expanded from 1.59% in R_1a to 3.49% in OL. *Ferruginibacter* showed modest growth during reclamation, peaking in R_2a with a 2.17% increase compared to OL. Additionally, some bacterial taxa exhibited varying correlations with the soil environment across different years (Figure 3). Members of the *Planktonophage*, *Nitrospiraea*, and Unclassified Bacteria phyla were present in very low abundance in all soil samples.

### 3.4. Diversity and PCA Analysis of Soil Bacterial Communities in Different Reclamation Years

Significant social variations were observed among the five regions, with R_2a displaying weaker systematic classification characteristics. Community richness, as indicated by the Shannon, Sobs, Heip, and Chao indices, exhibited a consistent pattern across all treatments, with R_6a having the highest value (Figure S2). In contrast, the Simpson and Coverage indices peaked in R_2a and were lowest in R_4a and R_1a, while the Ace index reached its lowest value in R_2a. The Shannon diversity index, reflecting relative species richness, ranged from 7.78 to 8.56. Diversity showed a decreasing pattern followed by an increase, eventually stabilizing with progressing treatment years. Heip’s diversity and homogeneity consistently exhibited low values in all samples. The sparsity curves indicated a trend toward stabilization for all soil samples (see Figure S2h). 

**Figure 4:**
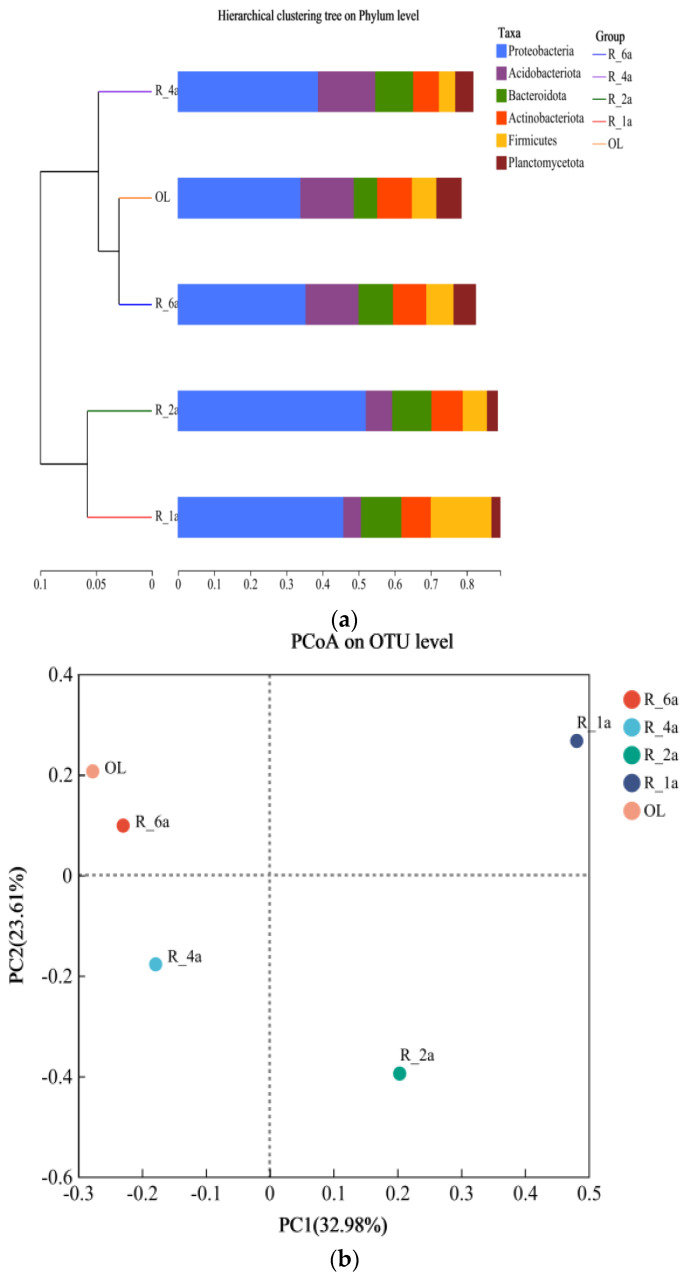

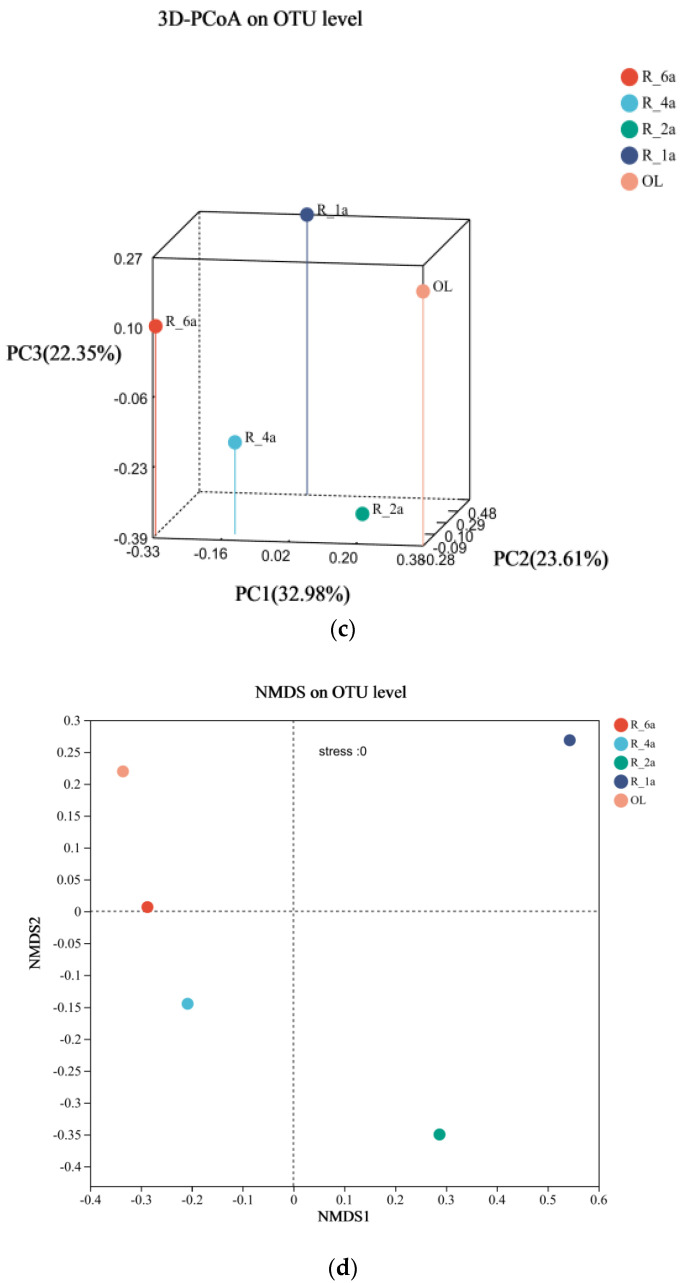
(**a**) Hierarchical clustering analysis of bacterial community samples using unweighted UniFrac distances to assess similarity among treatments; (**b**) PCoA diversity analysis at the OTU level, illustrating dispersion among all treatments; (**c**) 3D-PCoA, multidimensional PCA analysis at the subgroup level, comparing bacterial communities under different treatment factors; (**d**) Confirmation of bacterial community similarity among different treatments through NMDS analysis.

Average Sample Level Clustering analysis identified two distinct clusters (Figure 4a). One cluster included R_1a and R_2a, while the other comprised OL, R_6a, and R_4a. This suggests a high degree of similarity in bacterial communities among OL, R_6a, and R_4a, which clustered together. Beta diversity revealed significant differences among the five reclamation years. PCoA (Figure 4b,c) illustrated that two major axes explained 56.59% of the overall bacterial community variation. Notably, R_2a and R_4a exhibited closely clustered bacterial diversity. NMDS analysis confirmed the similarity of bacterial communities across all treatments, represented by a stress value of <0.05, indicating a reliable representation (Figure 4d). As expected, NMDS analysis revealed changes in bacterial community diversity in different soil samples over time. The outcomes of ANOSIM (Figure S4) indicated significant distinctions (*p* = 0.112, number of permutations = 999; R2 = 0.74903; Pr = 0.1).

### 3.5. Correlation between Soil Physicochemical Properties and Microorganisms in Different Reclamation Years

**Figure 5:**
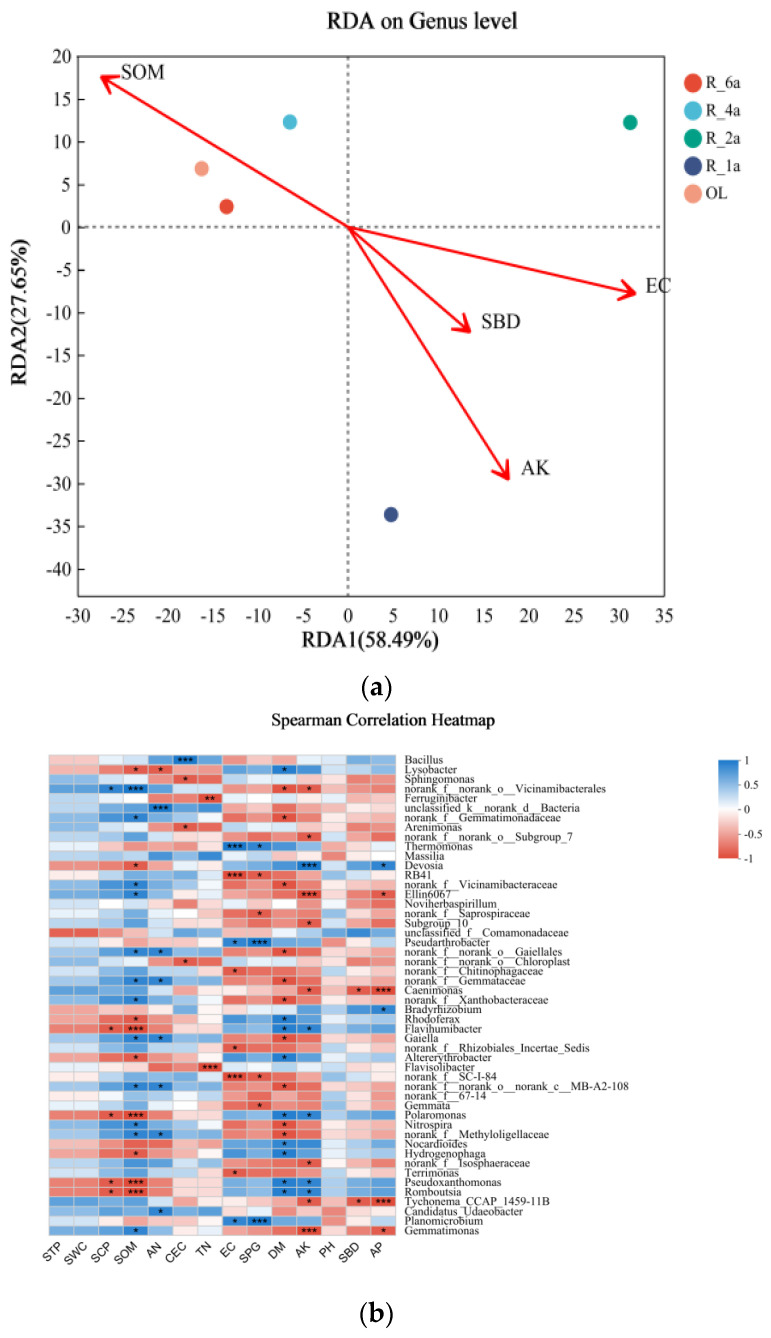

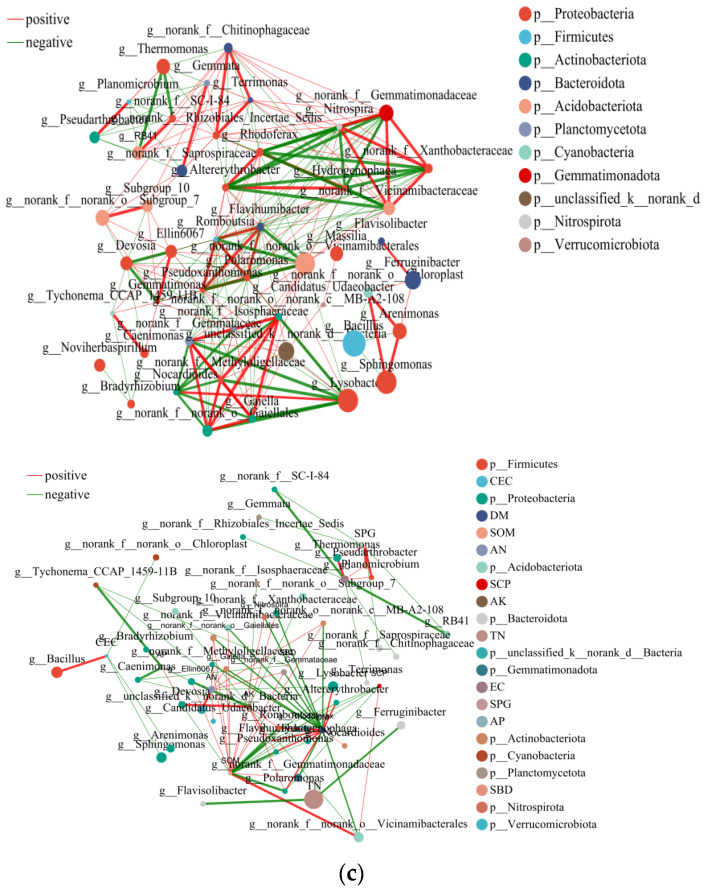
(**a**) RDA analysis shows correlations between soil properties and dominant bacterial genera. (**b**) Heatmap illustrating correlations between soil bacterial taxa and soil physicochemical properties at the genus level or higher within each soil type. Figure Note: The X-axis and Y-axis denote environmental factors and species, respectively. * 0.01 < *p* ≤ 0.05, ** 0.001 < *p* ≤ 0.01, *** *p* ≤ 0.001. (**c**) Species correlation network map depicting species correlations at each taxonomic level under specific environmental conditions. Node size, colors, line colors, and line thickness indicate species abundance, positive/negative correlations, and correlation strength, respectively.

VIF analysis uncovered notable multicollinearity among various soil properties, detailed in Table S1. Specifically, SPG, pH, TN, EC, CEC, SWC, HN, SOM, AP, SCP, STP, and AK demonstrated interrelated covariance. RDA analyses showcased distinct impacts of soil properties on bacterial communities, with SOM being more influential in long-term restoration soils and SBD having a lesser effect. The statistical significance of these relationships was affirmed through Mantel’s test across standard axes (*p* = 0.001), depicted in Figure 5a. Quantitative insights into the effects of soil properties on bacterial composition at the genus level are summarized in Table S2. RDA1 and RDA2, representing 86.14% of the variance, emphasize the significant role of soil properties in shaping the bacterial community. Key determinants affecting bacterial composition include soil AK (*p* = 0.0083), TN (*p* = 0.0333), SOM (*p* = 0.0417), EC (*p* = 0.1083), and SBD (*p* = 0.55). The program effectively distinguishes OL from the four reclamation age systems, as shown by distinct colored squares. Additionally, the Heatmap plot (Figure 5b) illustrates the correlation between soil properties and bacterial genus, highlighting how the dominant genus responds differently to variations in soil properties. Regression analysis unveiled significant correlations between soil properties (AK, TN, SOM, EC, and SBD) and specific genera (Figure S5). Co-occurrence network diagrams reflect the interactions between species in the sample, including *Verrucomicrobiota*, *Bacteroidota*, *Proteobacteria*, *Gemmatimonadota*, *Chloroflexi*, *Firmicutes*, *Actinobacteriota*, *Planctomycetota*, and more (Figure 5c).

### 3.6. Microbial Evolution and Potential Functional Shifts in Soils of Different Reclamation Years

**Figure 6:**
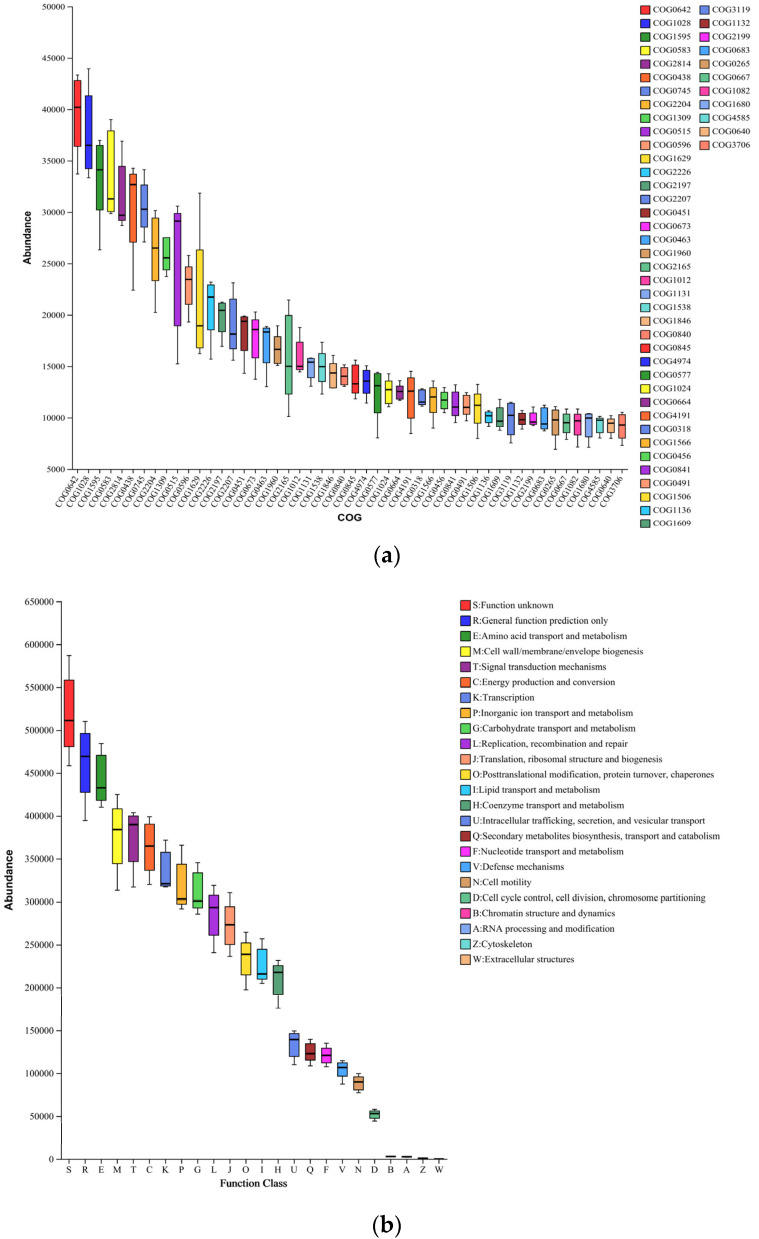

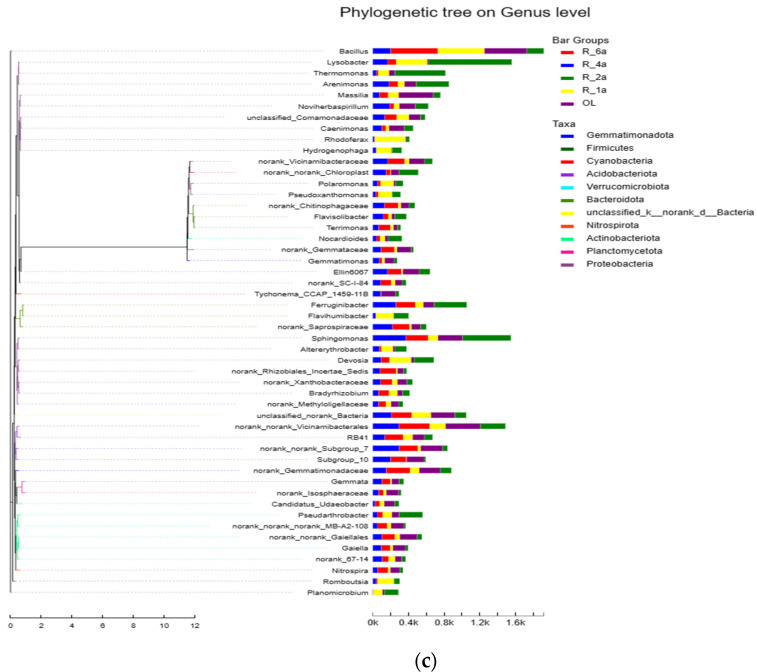
(**a**) COG Abundance Box plot depicting functional abundance statistics. Note: Horizontal coordinates represent COG function numbers, and vertical coordinates represent functional abundance. (**b**) Box Plot showcasing COG Functional Classification Statistics. Note: Horizontal coordinates represent COG secondary function numbers, and vertical coordinates represent functional abundance. (**c**) Phylogenetic Tree. The left side displays the phylogenetic evolutionary tree, with species represented by branches. Branches are color-coded to indicate higher taxonomic levels, and branch length represents evolutionary distances between species, signifying their degree of difference. The right-side bar chart indicates the proportion of reads from various subgroups.

Phylogenetic analyses were performed on major taxa from the microbial communities. High-abundance OTUs were selected as representatives for precise species annotation through comparison with the NCBI database. Phylogenetic trees were constructed using a maximum likelihood approach with 500 bootstrap replicates based on 16S rRNA gene sequences. Additionally, PICRUSt, a cost-effective tool, was utilized for predicting bacterial functions from 16S rRNA gene data. This approach helps us understand soil bacteria’s adaptation to different reclamation times on reclaimed land. We extracted information about individual COGs from the eggNOG database, including their functional attributes, resulting in functional abundance profiles (Figure 6a,b). The majority of the predicted sequences were associated with functions classified as “unknown function”, “genus-specific function prediction”, “amino acid transport and metabolism”, “cell wall/membrane/envelope biogenesis”, “signaling mechanisms”, and “energy production and conversion”. Notably, the transcription category showed a higher abundance of histidine kinases and dehydrogenase reductases. Using data from the KEGG database, we accessed information on KO, Pathway, and EC, allowing us to calculate functional class abundance based on OTU abundance (Figure S6). Regarding pathways, PICRUSt offered three metabolic pathway-related information levels. We scrutinized the predicted functions of soil bacterial community evolution and environmental adaptation (Figure 6c). Notably, sequences linked to *Bacillus*, *Lysobacillus*, *Aeromonas*, *norank_norank_Vicinamibacterales*, and *Sphingomonas* showed substantial increases in abundance across the recovery years.

## 4. Discussion

Large-scale mining significantly harms soil integrity, with waste infiltration causing soil infertility and degradation, particularly in sensitive regions like the Tibetan Plateau, known for its cold climate, alpine permafrost, and delicate ecosystem [27]. High rainfall in mining areas worsens soil and water erosion, accelerating soil organic matter and nutrient loss, disrupting soil structure, and hampering plant growth and recovery. Therefore, it is crucial to improve soil structure and monitor nutrient and microorganism changes during vegetation restoration for effective mining site ecological rehabilitation. The interplay between soil microorganisms and plants is bidirectional and influenced by the inter-root microbial community composition. Environmental stressors, such as soil property changes, directly affect this community’s composition and, consequently, plant performance [28,29]. Plants play a vital role in shaping inter-root microbial communities by releasing root secretions in response to environmental changes [30,31]. In alpine mining ecosystems, intricate and synergistic interactions between plant communities and soil microorganisms enrich soil nutrients and organic matter while inhibiting further metal sulfide oxidation [32,33]. In copper extraction and sulfur production, abundant metal sulfides are produced, with microorganisms being vital catalysts in transforming polymetallic sulfides. Studying soil microorganisms is crucial, as they, despite their limited presence, show remarkable sensitivity to soil changes, significantly impacting ecosystem functions [34]. Soil properties, such as nutrients (e.g., SOM, TN, AP), pH, salinity, water capacity, and organic matter nature, significantly shape bacterial community composition. Our findings underscore the strong link between key bacterial phyla and genus with SOM, in line with prior studies [35]. Reclaimed soil showed increased pH over time compared to the original state (OL). This is due to improved soil structure and microbial community, which resulted from the gradual incorporation of organic matter. The R_6a soil’s physicochemical properties closely resembled the original state (OL), with increased plant root and humus content, including SOM, HN, and TN, surpassing other treatment groups. These factors collectively played a pivotal role in pH regulation. Local factors like grazing-related fecal deposition, trampling compaction, selective herbivory, grassland regeneration, and regional rainfall patterns also influenced soil pH. It is crucial to emphasize that soil bacterial communities are highly responsive to environmental changes. Prior studies highlight the pivotal role of environmental factors, particularly soil pH, in shaping soil bacterial communities’ composition and diversity [36,37]. In our study, we observed a clear correlation between soil pH and the bacterial community. Over the years of reclamation, soil pH gradually decreased, leading to the selection and stabilization of bacterial species. Bacillus became the dominant genus in response to neutral soil conditions, emphasizing the impact of soil acidity and alkalinity on the microbial community. Spatial variations in soil conductivity significantly affect plant and animal activities, indicating soil salt concentration and moisture status, which are ecologically important. The decline in soil EC, attributed to organic matter’s buffering effect, differentiates it from control soils [38]. Higher regional rainfall leaches soluble salts, mainly affecting extended reclamation (R_6a and R_4a), while natural turf with established roots is more resistant to soil washout. Soil bulk density, porosity, and moisture content reveal valuable insights into soil structure, permeability, and water conservation capacity. SBD, reflecting soil structure and permeability, remained relatively stable, improving over extended reclamation. However, these values still differed from those observed in the original natural state (OL). The superior soil quality in natural turf is attributed to richer soil nutrients and diverse microbial populations, enhancing texture, structure, aeration, hydration, and water retention. As presented in Table 1, OLs pH was close to neutral, while EC and CEC levels were intermediate. Notably, smaller SBDs and larger SWCs were observed, indicating favorable soil porosity and field water-holding capacity. Additionally, HN and SOM were at optimal levels, ensuring an ample supply of nutrients for grass species, thus confirming the sustainability of the restoration effort. In contrast, other treatments display SBD variations due to the absence of soil sifting in previous ecological restoration, leading to higher SBD. The increased soil weight in 2022 is linked to fresh construction and compaction from rainfall. TSP is pivotal for soil functionality, with around 50% porosity being the ideal threshold for sufficient oxygen and water movement in the soil. Natural turf soils maintain optimal porosity levels, while other reclaimed areas show an increasing trend in soil porosity over time. SWC is crucial, with an ideal threshold exceeding 20%. Excessive water-holding capacity can reduce soil aeration, negatively impacting microbial activity and plant growth [39].

Cu, Zn, Pb, Cr, and Hg content falls below risk screening values, indicating low soil contamination risk in agricultural land. However, As, Cd, and Ni, exceeding risk screening values but below risk control values, suggest potential contamination, impacting edible agricultural product quality and safety standards. Initial heavy metal reductions result from early soil restoration measures. These measures initially reduce heavy metal concentrations in the soil. Subsequent years, however, see an increase due to plant introductions in the restoration process. Initially, young plants have limited metal absorption capacity during early growth and root development. As plants mature, they gradually accumulate more metals. To ensure safe utilization in the future, agronomic control and soil improvement are crucial. Robust soil and plant monitoring is needed. Prolonged reclamation has positively impacted soil, as seen in physicochemical indicators, heavy metals, and nutrient levels. Overall, soil improvement is evident, despite reduced AP and AK content. Reduced nutrient levels may impact later-stage grass growth, highlighting the importance of proper fertilization [40]. Improved physicochemical soil quality likely created a more favorable environment with increased active nutrients and better structure. In the short term, this boosted microbial diversity (Figure 2a) and caused significant microbial composition changes in restored soils compared to control soils. This increased the number of dominant genera and species. Certain bacterial taxa, such as uncultivated *Cryptococcaceae*, *WCHB1-32*, *Luteimonas*, *Cellvibrio*, *Pseudomonas*, *Brevundimonas*, *Romboutsia*, *Pseudoxanthomonas*, *Flavihumibacter*, and *Bradyrhizobium,* appeared in higher abundance in the restored soils but had negligible representation in the control soil (Figure S1). Conversely, some bacterial taxa, including *Lysobacter*, *Ferruginibacter*, *Arenimonas,* and *Thermomonas,* exhibited low relative abundance in the restored soil but increased in abundance in the control soil. Microbial population shifts in degraded soils indicate that certain bacteria thrive with extended reclamation, influenced by changing soil properties. For instance, increased soil organic matter promotes bacteria like Bacillus and Aspergillus, which excel at organic matter decomposition. *Bacteria* such as *Cellvibrio*, adept at cellulose decomposition, may also proliferate. Longer reclamation stabilizes soil properties, reducing diseases and populations like *Lysobacter*. *Lysobacter* is essential for biocontrol, inhibiting pathogenic microorganism growth, and mitigating soil-borne plant diseases. It also aids in organic matter decomposition, releasing nutrients for plant uptake, reducing contamination, and improving soil structure [41]. Such studies are essential for understanding microbial structure and dynamics, given the significant role of microbial species interactions [42]. In our study area, soil bacterial taxa (e.g., *Bacillus*, *Lysobacter*, *Abditibacterium*, and *Bryobacter*) in various reclamation years showed a preference for soil nutrients (AP, AK), porosity, and pH (Figure 5b), thriving predominantly in restored soils. Conversely, *Sphingomonas* and *Ferruginibacter* exhibited strong negative correlations with these factors. In the short term, time seems to promote the development and homogenization of certain soil bacterial taxa in restored soils, while control soils become more dominant with extended exposure to time (Figure 6c). Extensive research has demonstrated that soil microbial bacilli are abundant and diverse, playing a pivotal role in soil formation and nutrient provision. 

The top five bacterial genera across treatments included *Sphingomonas*, *Bacillus*, *Lysobacter*, *norank-f-norank-o-Vicinamibacterales*, and *Ferruginibacter,* playing a pivotal role in soil enhancement. Surprisingly, *Sphingomonas* and others were found in high-altitude, cold regions, likely due to yaks, sheep, soil composts, and manure sources. *Sphingomonas* aids plant survival in saline areas by producing catalase, which improves water retention and regulates soil structure [43]. In R_2a treatment, EC and *Sphingomonas* peaked. With more reclamation years, both EC and *Sphingomonas* gradually decreased and stabilized. *Bacillus*, with its nitrogen-fixing enzymes and phosphate-solubilizing capabilities, is abundant in early restoration due to its adaptable spore-forming characteristics [44,45]. *Bacillus* excels at water retention, preventing soil nutrient and water loss through polyglutamate formation. However, *Bacillus* content was lower in the R_2a and R_4a treatments, leading to reduced soil TN and HN levels. *Lysobacillus* plays critical roles in ecosystems, efficiently decomposing organic matter, improving soil structure, porosity, permeability, water retention, and detoxifying soil pollutants for soil purification [43]. In the initial reclamation stages, *Bacillus* was prominent, notably in the R_2a treatment when soil quality was poor. With years of reclamation, *Bacillus* declined, but organic matter, water retention, and soil structure improved. *Ferruginibacter*, vital for Cordyceps sinensis growth, remained throughout the reclamation stages, explaining its abundance in Qinghai. These bacteria play a key role in organic matter degradation, as evidenced by dilution curves showing their high abundance and uniformity in soil microflora. The second year of restoration had the most bacterial species, but the dominance percentage decreased due to changing microbial activity over time. *Proteobacteria* consistently dominate reclaimed high-altitude mining sites in Qinghai–Tibet, as in previous studies [46,47]. In the original landscape (OL), *Proteobacteria*, *Acidobacteriota*, *Bacteroidota,* and *Actinobacteriota* proportions resemble those in R_6a, indicating that land reclaimed for over six years is near its natural state. This shift is due to changes in nitrogen content, soil organic matter, vegetation, and agricultural activities, all influential factors for soil microbial communities [48,49]. *Proteobacteria* excel at adapting to nutrient-poor, arid environments by efficiently degrading polysaccharides. They thrive in arid conditions, forming symbiotic relationships with plants and facilitating inter-root nitrogen fixation [50,51]. The mutualistic relationship between plants and *Proteobacteria* benefits both, with the plant gaining nitrogen and the *Proteobacteria* receiving organic compounds and nutrients. This relationship is vital for plant growth and contributes to higher soil nitrogen content. In the initial restoration years, *γ-Proteobacteria* like *Lysobacter* increased while *β-Proteobacteria* decreased, revealing a notable trend in microbial abundance [44]. *Bacteroidota*, frequently co-occurring with *Firmicutes*, *Proteobacteria,* and *Actinobacteria*, play a key role in the root zone [52], particularly in semi-arid environments where they decompose organic compounds and transform them into more degradable forms. This enhances soil microbial community degradation processes. *Proteobacteria* and *Firmicutes* are notable for their functions in soil remediation, including antibiotic production, organic matter decomposition, and enzyme production. Their degradation capabilities benefit the breakdown of organic matter, providing nutrients to plants and other microorganisms. Firmicutes’ abundance is typically seen in heavy metal-contaminated soil areas, and they excel at utilizing stable organic compounds, promoting soil health and balance [6,43,47]. The *Firmicutes* phylum initially increased in soil R_1a but later declined in R_2a and R_6a, possibly due to competition with other bacterial groups. Acidobacteriota’s presence grew with reclamation time, inhibiting harmful bacteria, enhancing food nutrition, and reducing microbial species. *Actinobacteriota* possess genes for breaking down recalcitrant organic compounds, which are valuable in stressed soils [53]. *Chloroflexi* and *Acidobacteriota* are associated with plant organic matter decomposition [54]. A small proportion of *Desulfobacterota*, important in biogeochemical cycles and environmental remediation, was also observed. These dominant groups have been noted in other agro-soil ecosystems [55,56]. Different bacterial communities have adapted to the alpine mines of Qinghai–Tibet [57].

Bacterial diversity in reclaimed soil initially increased, with diversity indices surpassing the control at various reclamation times. This higher diversity results from changes in the soil environment influenced by physicochemical properties and root secretions. Interventions and planting maintenance in different reclamation periods disrupt existing bacterial communities, fostering new ones. A mutual interaction exists between soil microorganisms and physicochemical properties. The observed positive correlation between physicochemical characteristics and soil microorganisms, mainly in R_6a and to some extent in R_2a, R_4a, and R_1a, suggests that the time-based remediation approach positively improved soil quality during its initial stages. Our analysis predicted soil bacterial community functions related to nutrient cycling and environmental adaptation (Figure 6). Positive correlations were observed between these functions and various soil reclamation years (Figure S6). However, it is important to interpret these predictions cautiously, considering the limitations of PICRUSt. Further macro-genomic studies are essential in later stages to comprehensively understand the relationship between bacterial function and soil properties. 

In conclusion, these studies highlight the positive influence of time-based treatments on soil physicochemical properties and bacterial diversity. Restored soil bacterial communities increased in both abundance and nutrient-releasing activity over control soils in the short term. The Qinghai–Tibet alpine mines harbor diverse bacterial adaptations, providing insights into temporal bacterial dynamics. This information can guide future management strategies for selecting effective measures.

## 5. Conclusions

In this comprehensive study of high-alpine mining reclamation, we found that extended reclamation time significantly improved soil physicochemical properties. Notably, it led to a reduction in soil conductivity, an increase in cation exchange capacity, capillary porosity, and stability index, along with elevated levels of soil organic matter and hydrolyzable nitrogen. These changes indicated a decline in soil salinity and an enhancement of organic matter, fostering greater microbial diversity. Environments with high nutrient levels and low salinity, particularly the R_6a reclamation site, demonstrated the most pronounced impact on soil bacterial diversity. Heavy metal contamination showed signs of self-healing over time. The dominant bacterial phyla included *Proteobacteria* > *Acidobacteriota* > *Bacteroidota* > *Actinobacteriota* > *Firmicutes*, with notable genera like *Bacillus*, *Lysobacter*, and *Sphingomonas.* Soil bacterial community diversity increased across all reclamation systems, with R_6a having the highest diversity. This highlights the importance of the number of years dedicated to vegetation restoration in enhancing soil bacterial diversity. In the second year of reclamation, a substantial decrease in soil available phosphorus (AP) was observed. The application of bacterial fertilizers, primarily consisting of Firmicutes, could be essential in replenishing effective phosphorus content and stimulating bacterial community growth. The influence of soil properties, including SOM, TN, AP, pH, EC, and SWC, played pivotal roles in shaping bacterial community composition. PICRUSt predictions indicated varying effects of different reclamation years not only on bacterial composition and diversity but also on key metabolic functions. While sequencing data and functional predictions provide valuable insights, caution should be exercised when drawing definitive conclusions. We recommend extensive, long-term investigations to gain a deeper understanding of how time influences the evolution of microbial communities and their functional dynamics.

## Figures and Tables

**Table 1:** Basic information about the plot.

Plot Number	Plot Name	Reclamation Period	Types of Reclaimed Vegetation	Aspect of Slope
R_1a(S1)	4242BP	1	*Elymus nutans*, *Poa crymophila cv Qinghai*, *Elymus sibiricus* L., *Festuca sinensis*, *Poa pratensis* L., *Floret alkali grass*	West
R_2a(S2)	4386BP	2	*Elymus nutans*, *Poa crymophila cv Qinghai*, *Elymus sibiricus* L., *Festuca sinensis*, *Poa pratensis* L., *Floret alkali grass*, *ryegrass*	west
R_4a(S3)	4230BP	4	*Elymus nutans*, *Poa crymophila cv Qinghai*, *Elymus sibiricus* L., *Festuca sinensis*, *rape flower*	west
R_6a(S4)	4420BP	6	*Elymus nutans*, *Poa crymophila cv Qinghai*, *Elymus sibiricus* L., *Festuca sinensis*	west
OL(S5)	4242YT	—	*Elymus nutans*, *Poa crymophila cv Qinghai*, *Elymus sibiricus* L., *Festuca sinensis*, *Kobresia pygmaea*, *Kobresia humilis*	west

R_1a: the reclamation time is one year; R_2a: the reclamation time is two years; R_4a: the reclamation time is four years; R_6a: the reclamation time is six years; OL: unspoiled natural grassland.

**Table 2:** Basic physicochemical properties (Mean, n = 3).

Treatment	Unit	R_1a	R_2a	R_4a	R_6a	OL
pH	/	7.91 ± 0.01 ab	7.55 ± 0.55 b	8.00 ± 0.03 a	7.91 ± 0.06 ab	7.04 ± 0.11 c
EC	μs·cm^−1^	205.97 ± 19.07 b	272.33 ± 29.33 a	92.97 ± 2.47 d	77.00 ± 60.00 d	124.50 ± 2.50 c
CEC	cmol·kg^−1^	18.43 ± 2.84 ab	12.30 ± 1.18 b	13.17 ± 0.02 b	27.98 ± 6.96 a	16.75 ± 0.04 ab
SBD	g·cm^−3^	1.66 ± 0.07 a	1.56 ± 0.06 a	1.60 ± 0.01 a	1.61 ± 0.02 a	1.16 ± 0.11 b
SPG	g·cm^−3^	2.65 ± 0.02 a	2.67 ± 0.01 a	2.59 ± 0.04 a	2.62 ± 0.02 a	2.63 ± 0.01 a
STP	%	37.39 ± 0.02 b	41.67 ± 0.02 b	38.35 ± 0.10 b	38.45 ± 0.80 b	55.72 ± 0.04 a
SCP	%	23.63 ± 0.09 b	30.50 ± 0.01 ab	26.50 ± 0.02 ab	33.50 ± 0.04 ab	42.13 ± 0.03 a
SWC	%	19.46 ± 1.80 b	21.42 ± 1.20 b	19.75 ± 0.80 b	19.88 ± 0.60 b	38.17 ± 1.10 a
TN	mg·kg^−1^	1220.00 ± 58.8 a	1020.00 ± 49.98 b	1150.00 ± 56.35 ab	1200 ± 58.80 a	1220.00 ± 60.00 a
DM	%	99.00 ± 2.60 a	99.00 ± 1.80 a	98.70 ± 2.50 a	98.20 ± 3.20 a	98.20 ± 3.60 a
SOM	g·kg^−1^	17.70 ± 0.50 c	21.70 ± 0.50 b	31.40 ± 0.30 ab	41.40 ± 0.20 a	43.20 ± 0.60 a
HN	mg·kg^−1^	81.10 ± 4.87 b	78.40 ± 4.70 b	81.00 ± 4.86 b	174.00 ± 10.44 ab	243.00 ± 14.58 a
AP	mg·kg^−1^	19.60 ± 0.80 a	11.20 ± 0.90 ab	3.20 ± 0.70 b	12.40 ± 0.50 a	1.90 ± 0.70 b
AK	mg·kg^−1^	150.00 ± 4.00 b	93.00 ± 4.00 ab	58.00 ± 2.00 a	60.00 ± 50.00 a	50.00 ± 50.00 a

Note: Different lowercase letters after the data of different treatments in the same column in the table indicate significant differences (*p* >0.05).

**Table 3:** Soil partial heavy metal index (mean, n = 3).

Treatment	unit	R_1a	R_2a	R_4a	R_6a	OL
Cu	mg·kg^−1^	28.00 ± 0.84 b	87.00 ± 2.61 ab	57.00 ± 1.71 a	50.00 ± 1.50 a	39.00 ± 1.17 c
Cr	mg·kg^−1^	78.00 ± 0.70 b	167.00 ± 1.50 a	292.00 ± 2.63 ab	174.00 ± 1.57 a	166.00 ± 1.49 a
Ni	mg·kg^−1^	52.00 ± 0.62 c	131.00 ± 1.57 ab	429.00 ± 5.2 b	259.00 ± 3.11 a	122.00 ± 1.46 c
Zn	mg·kg^−1^	76.00 ± 1.30 a	99.00 ± 1.10 a	87.00 ± 1.20 b	84.00 ± 1.30 a	93.00 ± 1.10 ab
Pb	mg·kg^−1^	14.00 ± 0.41 a	24.00 ± 0.70 a	14.00 ± 0.41 a	18.00 ± 0.52 a	16.00 ± 0.46 a
Cd	mg·kg^−1^	0.05 ± 0.02 a	0.05 ± 0.02 a	0.07 ± 0.01 a	0.03 ± 0.01 b	0.04 ± 0.01 b
As	mg·kg^−1^	17.60 ± 0.51 a	32.00 ± 0.19 ab	12.40 ± 0.07 a	23.60 ± 0.14 a	14.20 ± 0.41 a
Hg	mg·kg^−1^	0.10 ± 0.003 a	0.43 ± 0.001 b	0.113 ± 0.002 ab	0.054 ± 0.001 b	0.05 ± 0.002 b

Note: Different lowercase letters after the data of different treatments in the same column in the table indicate significant differences (*p* >0.05).

## Data Availability

Data are contained within the article.

## Supplementary Materials

The following supporting information can be downloaded at: https://www.mdpi.com/article/10.3390/microorganisms12010041/s1.
