# Supplementary material for: Impact of Ecological Restoration on the Physicochemical Properties and Bacterial Communities in Alpine Mining Area Soils
Source: Microorganisms. 2023 Dec 25;12(1):41. doi: 10.3390/microorganisms12010041 (PMC10818615; doi:10.3390/microorganisms12010041)
Supplement: Supplementary file 1 [file microorganisms-12-00041-s001.zip › microorganisms-2759609-supplementary.pdf]

Article

Impact of Ecological Restoration on the Physicochemical Properties and Bacterial Communities in Alpine Mining Area Soils

Lingjian Kong, Lin Zhang, Yingnan Wang and Zhanbin Huang *

School of Chemical and Environmental Engineering, China University of Mining and Technology (Beijing), Beijing 100083, China; ljkong2023@163.com (L.K.); wendyl0902@163.com (L.Z.); wangyingsouth@163.com (Y.W.)

* Correspondence: 108742@cumtb.edu.cn

Text S1 Presentation of soil properties in the study area

Copper mining at Qinghai West Minster commenced in the early 21st century but was concluded a decade ago (Fig. 1). The primary soil-forming parent material at the mine site comprises alluvial and flood deposits, including residual-slope deposits. During the initial construction phase, a substantial volume of serpentine residue and raw soil was generated by stripping the mine during the ore extraction process. This serpentine soil is characterized by its barren nature, limited water retention capacity, and thin soil layer. Initially, the plan was to employ indigenous natural turf soil as the planting substrate for ecological revegetation. However, due to the limited availability of this native soil, an alternative land reclamation and restoration approach was investigated. This involved the utilization of the abundant serpentine present in the mining area to supplement the raw soil for the restoration process. To create the experimental soil mixture, serpentine, and raw soil were carefully blended in specific proportions to ensure uniformity. The experimental serpentine soil displayed the following physicochemical properties: pH 8.81, electrical conductivity (EC) 308.3 μs/cm, soil bulk density (SBD) 1.36 $g{\bullet}cm^{-1}$, soil organic matter (SOM) 1.5 $g{\bullet}kg^{-1}$, hydrolyzable nitrogen (HN) 5.73 $mg{\bullet}kg^{-1}$, available phosphorus (AP) 124 $mg{\bullet}kg^{-1}$, and available potassium (AK) 1800 $mg{\bullet}kg^{-1}$. In contrast, the raw soil used for the experiment exhibited the following physicochemical properties: pH 7.65, EC 293.7 μs/cm, SBD 1.38 $g{\bullet}cm^{-1}$, SOM 5.7 $g{\bullet}kg^{-1}$, HN 55.5 $mg{\bullet}kg^{-1}$, AP 312 $mg{\bullet}kg^{-1}$, and AK 3000 $mg{\bullet}kg^{-1}$. For our study, we selected five slopes in the discharge field, each representing different reclamation years, as illustrated in Fig. 1. These include R_6a: Slopes reclaimed in 2017, corresponding to year 6 of reclamation. R_4a: Slopes reclaimed in 2019, corresponding to year 4 of reclamation. R_2a: Slopes reclaimed in 2021, corresponding to year 2 of reclamation. R_1a: Slopes reclaimed in 2022, corresponding to year 1 of reclamation. OL: An unmined area featuring natural grassland, unaffected by mining activities in the mine site.

Text S2 Steps in the PCR reaction

The PCR reaction mixture consisted of 4 μL of 5× FastPfu buffer, 2 μL of 2.5 mM dNTPs, 0.8 μL of the upstream primer (5 uM), 0.8 μL of the downstream primer (5 uM), 0.4 μL of FastPfu polymerase, 0.2 μL of BSA, and 10 ng of template DNA, resulting in a total volume of 20 μL. Each sample was prepared in triplicate. The PCR program included an initial denaturation step at 95℃ for 3 minutes, followed by 29 cycles (denaturation at 95℃ for 30 seconds, annealing at 60℃ for 30 seconds, and extension at 72℃ for 45 seconds), with a final extension step at 72℃ for 10 minutes, and a storage temperature of 10 ℃. The PCR was conducted using an ABI GeneAmp® Model 9700 instrument. The PCR

products from the same sample were combined and subjected to 2% agarose gel electrophoresis. Subsequently, magnetic bead purification was carried out, and the purified products were quantified using a Quantus™ Fluorometer (Promega, USA). The mixtures were then prepared in the required proportions for sequencing each sample.

Text S3 Illumina sequencing involved a series of steps

Illumina sequencing involved a series of steps: 1) One end of the DNA fragment was paired with the primer base and anchored to the chip; 2) PCR synthesis used the DNA fragment as a template, with sequences on the chip serving as primers to synthesize the target DNA fragment on the chip; 3) After denaturation and annealing, the other end of the DNA fragment on the chip randomly paired with another nearby primer, forming a "bridge"; 4) PCR amplification generated DNA clusters; 5) The DNA amplicon was linearized into a single strand; 6) Modified DNA polymerase and dNTPs with four fluorescent markers were added to synthesize one base per cycle; 7) Laser scanning of the reaction plate's surface read the type of nucleotide polymerized in the first round of the reaction; 8) The "fluorescent group" and "termination group" were chemically cleaved to restore the 3' end's stickiness, enabling the continuation of polymerization with the second nucleotide; 9) Fluorescence signals collected in each round were statistically analyzed to determine the sequence of the template DNA fragments.

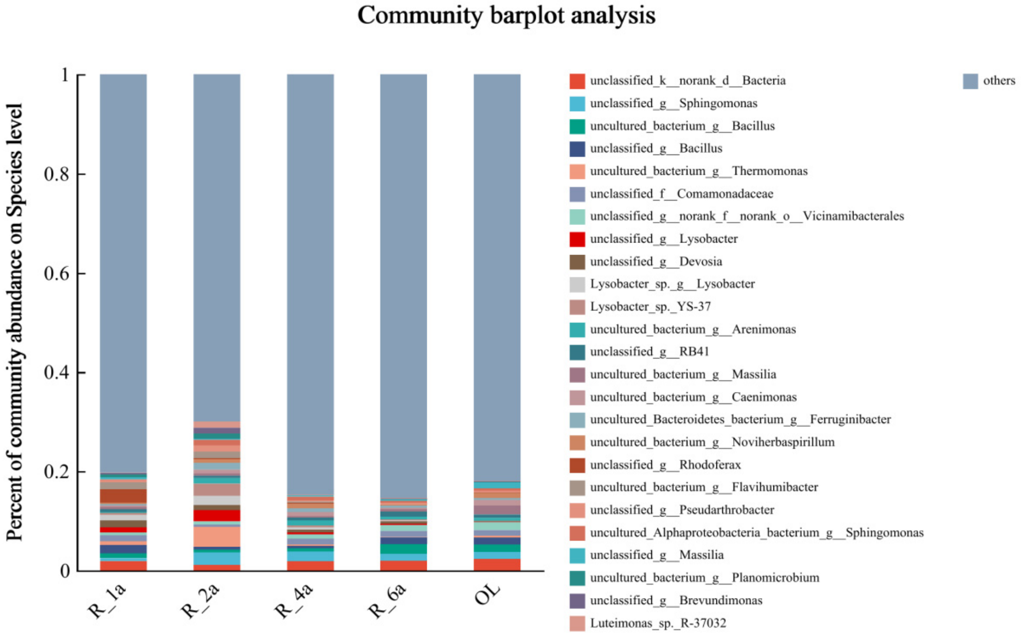

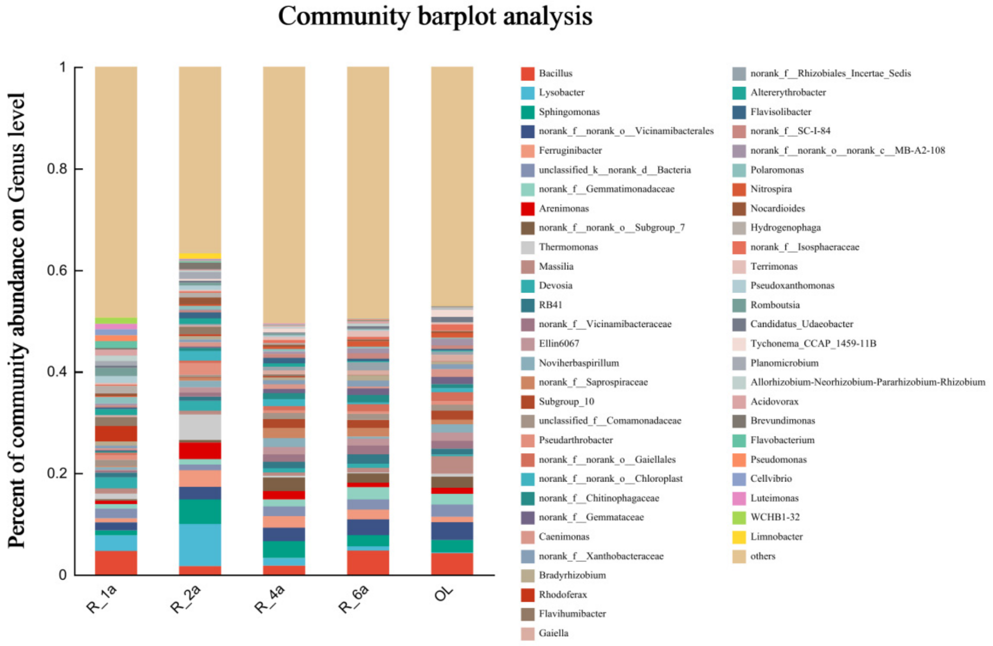

Figure S1. Composition of the soil bacterial communities of the different treatment groups at the genus and species level.

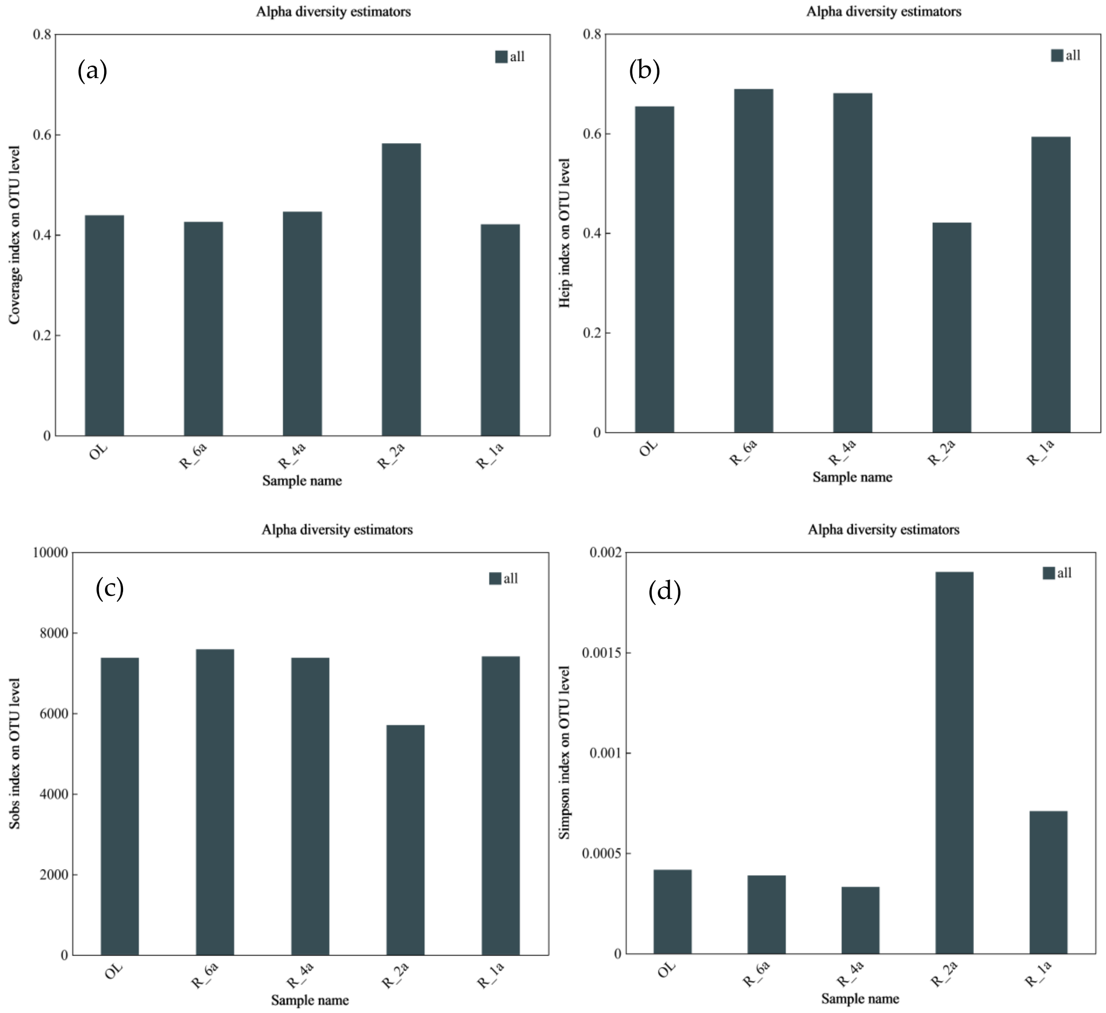

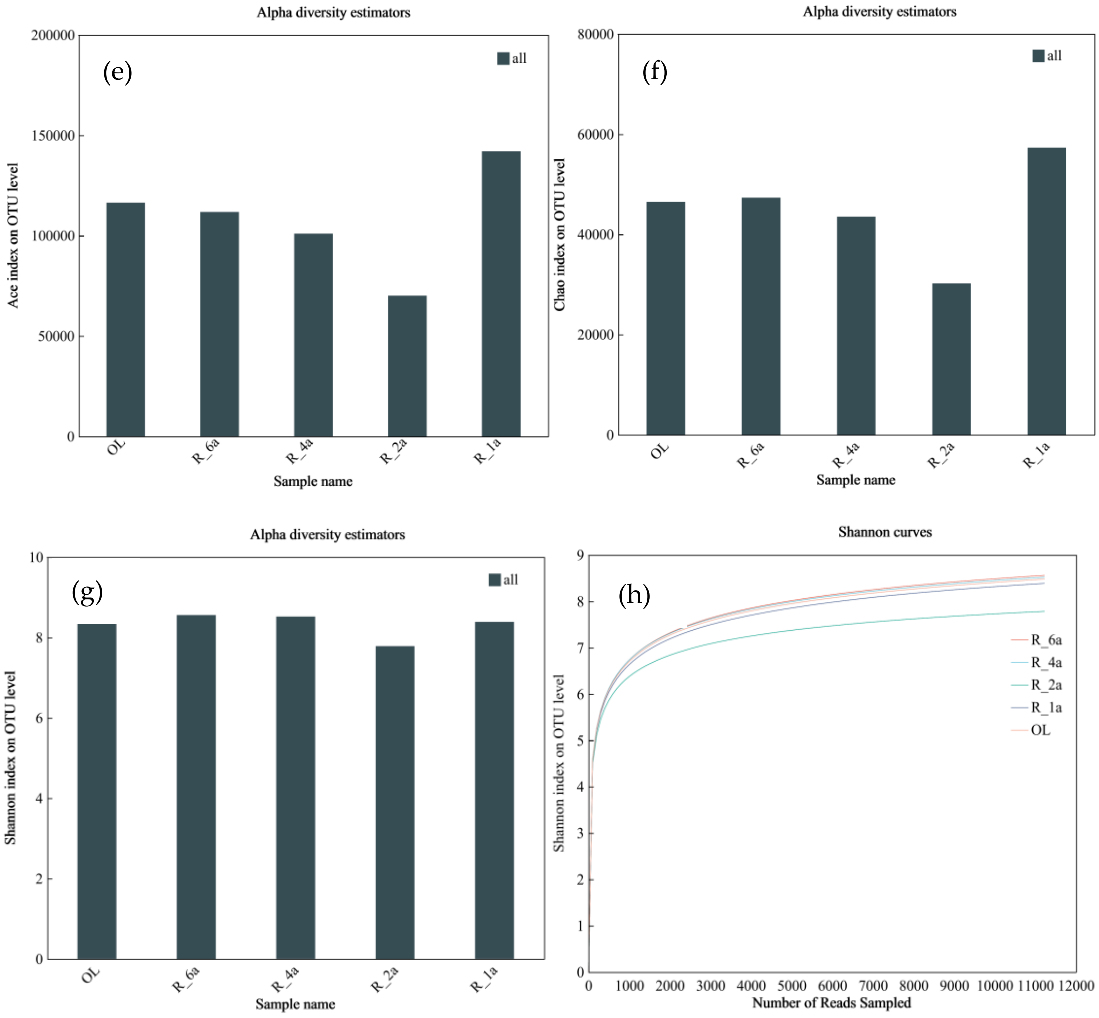

Figure S2. (a-g) soil microbial community diversity and composition Coverage, Heip, Sobs, Chao, Simpson, Shannon, Ace index; (h) Dilution curve, which shows whether the amount of data for this sequencing is sufficient or not based on whether the curve has reached a plateau or not using the Alpha diversity index value as a reference;.

(a)

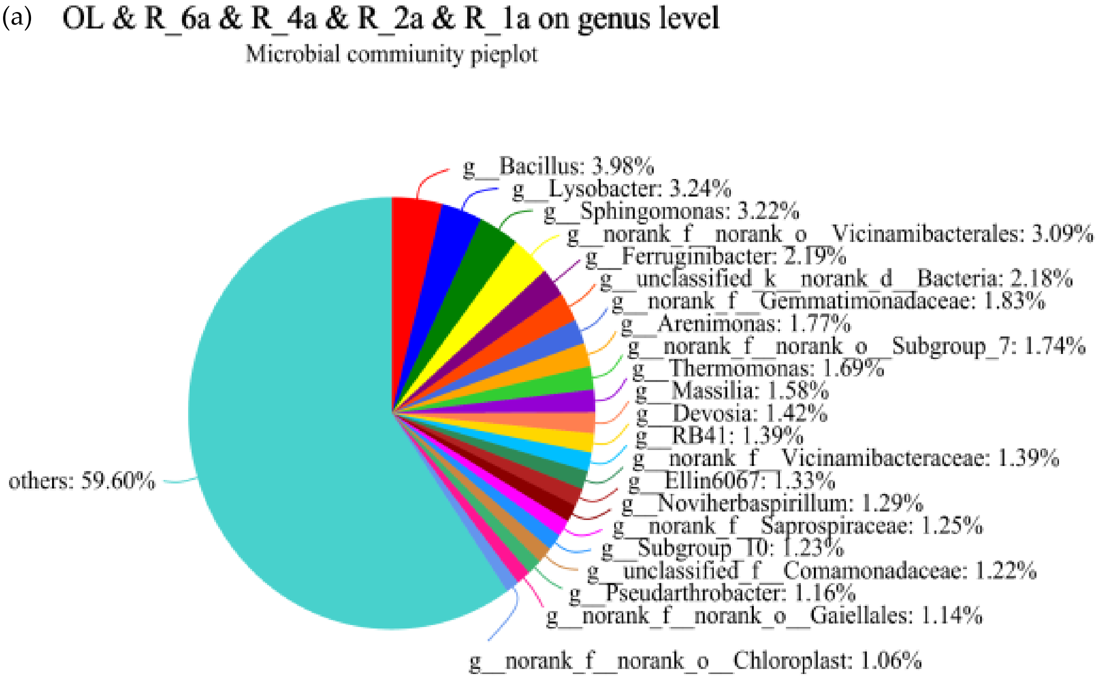

(b)

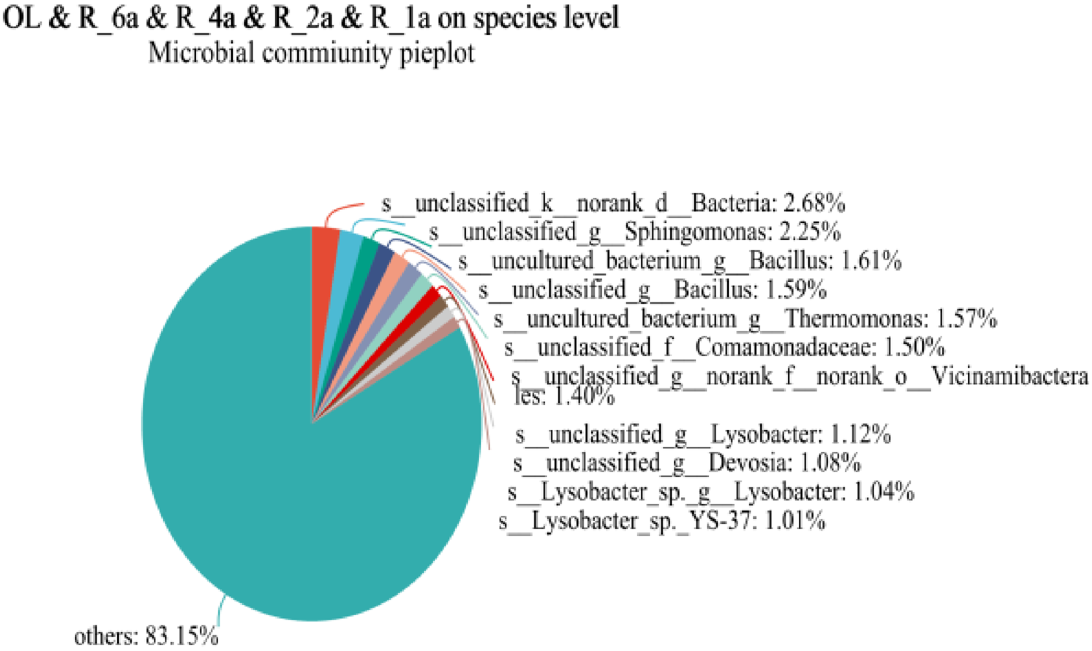

Figure S3. Pie charts showing the relative abundance of microorganisms in 5 different samples at the genus and species levels.

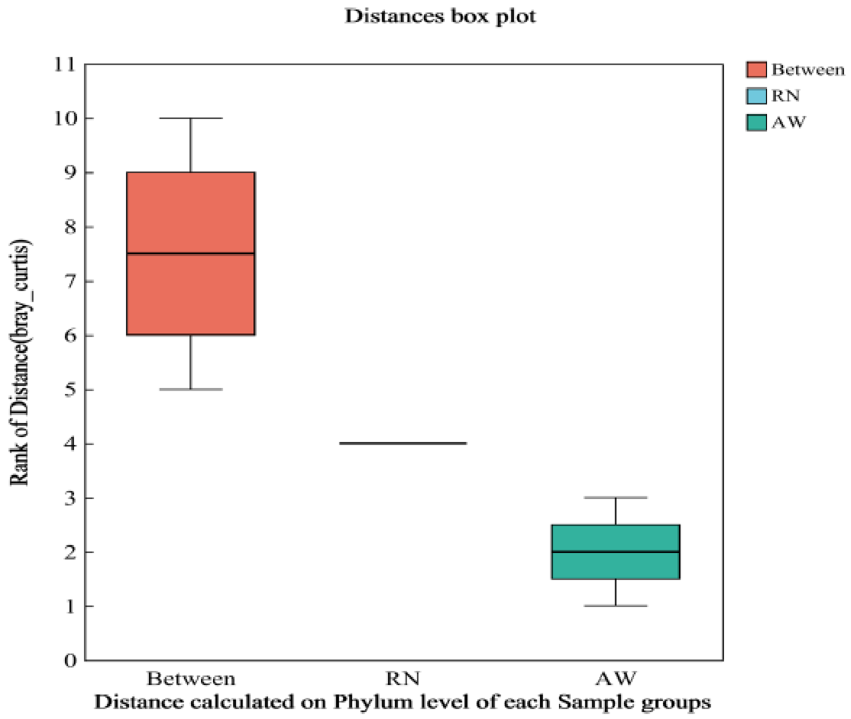

Figure S4. Distances calculated by phylum level for each sample group.

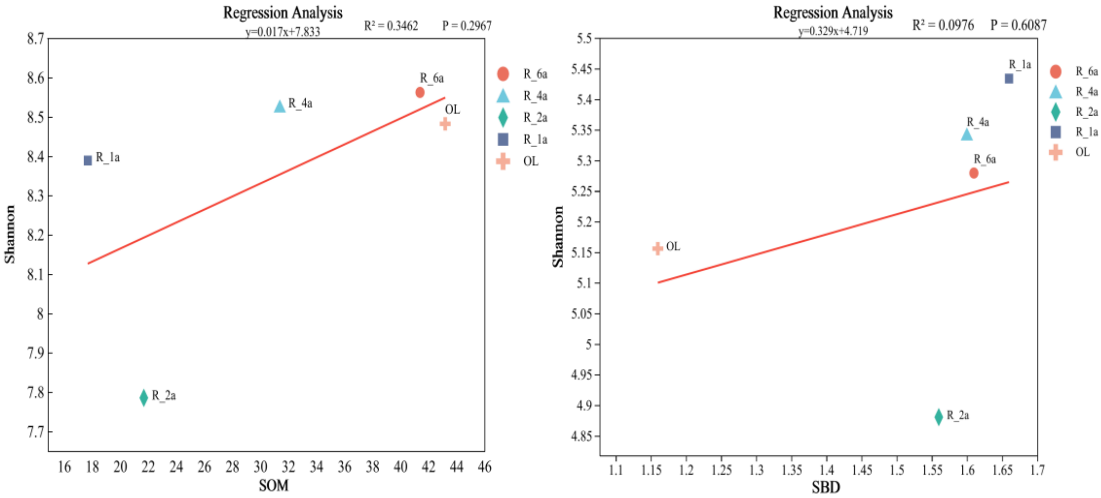

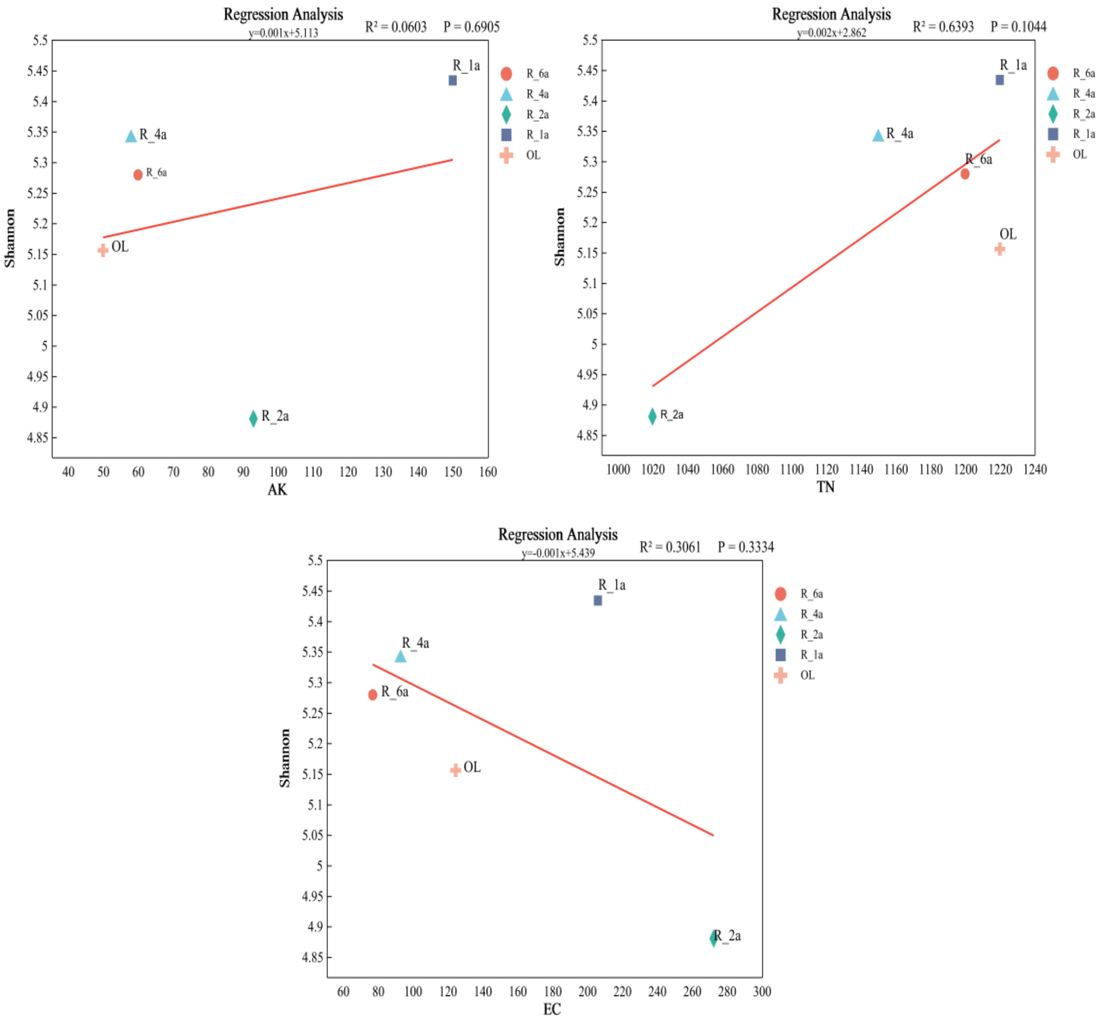

Figure S5. Based on the results of the PCoA analysis, the linear regression (Linear Regression), labeled with R^2, can be used to evaluate the relationship between the two. Where R^2 is the coefficient of determination, a ranked regression analysis plot of the proportion of variation explained by the regression line; .

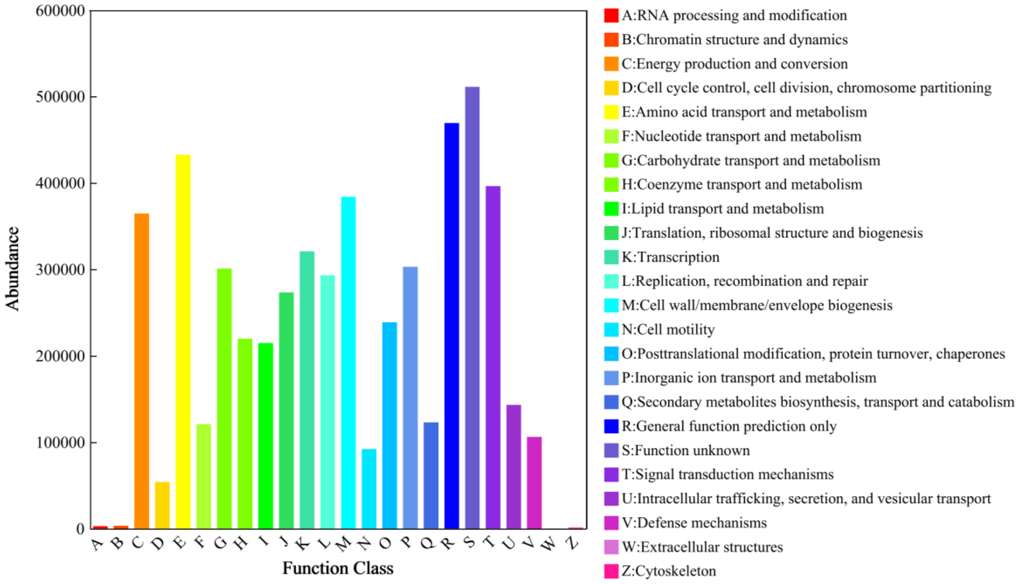
600000
500000
400000
300000
200000
100000
0
Abundance
A B C D E F G H I J K L M N O P Q R S T U V W Z
Function Class
A:RNA processing and modification
B:Chromatin structure and dynamics
C:Energy production and conversion
D:Cell cycle control, cell division, chromosome partitioning
E:Amino acid transport and metabolism
F:Nucleotide transport and metabolism
G:Carbohydrate transport and metabolism
H:Coenzyme transport and metabolism
I:Lipid transport and metabolism
J:Translation, ribosomal structure and biogenesis
K:Transcription
L:Replication, recombination and repair
M:Cell wall/membrane/envelope biogenesis
N:Cell motility
O:Posttranslational modification, protein turnover, chaperones
P:Inorganic ion transport and metabolism
Q:Secondary metabolites biosynthesis, transport and catabolism
R:General function prediction only
S:Function unknown
T:Signal transduction mechanisms
U:Intracellular trafficking, secretion, and vesicular transport
V:Defense mechanisms
W:Extracellular structures
Z:Cytoskeleton

(**a**)

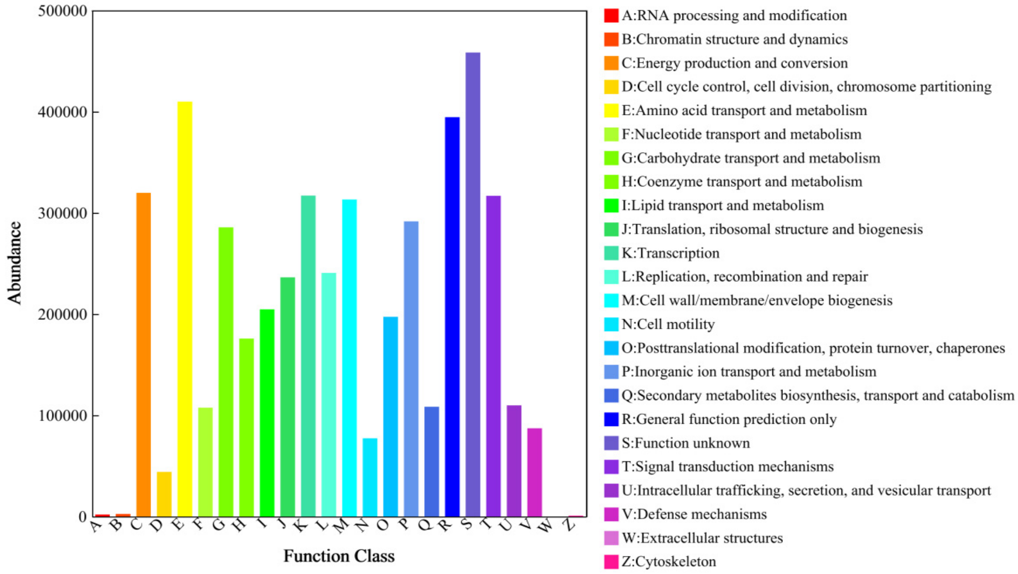
500000
400000
300000
200000
100000
0
Abundance
A B C D E F G H I J K L M N O P Q R S T U V W Z
Function Class
A:RNA processing and modification
B:Chromatin structure and dynamics
C:Energy production and conversion
D:Cell cycle control, cell division, chromosome partitioning
E:Amino acid transport and metabolism
F:Nucleotide transport and metabolism
G:Carbohydrate transport and metabolism
H:Coenzyme transport and metabolism
I:Lipid transport and metabolism
J:Translation, ribosomal structure and biogenesis
K:Transcription
L:Replication, recombination and repair
M:Cell wall/membrane/envelope biogenesis
N:Cell motility
O:Posttranslational modification, protein turnover, chaperones
P:Inorganic ion transport and metabolism
Q:Secondary metabolites biosynthesis, transport and catabolism
R:General function prediction only
S:Function unknown
T:Signal transduction mechanisms
U:Intracellular trafficking, secretion, and vesicular transport
V:Defense mechanisms
W:Extracellular structures
Z:Cytoskeleton

(**b**)

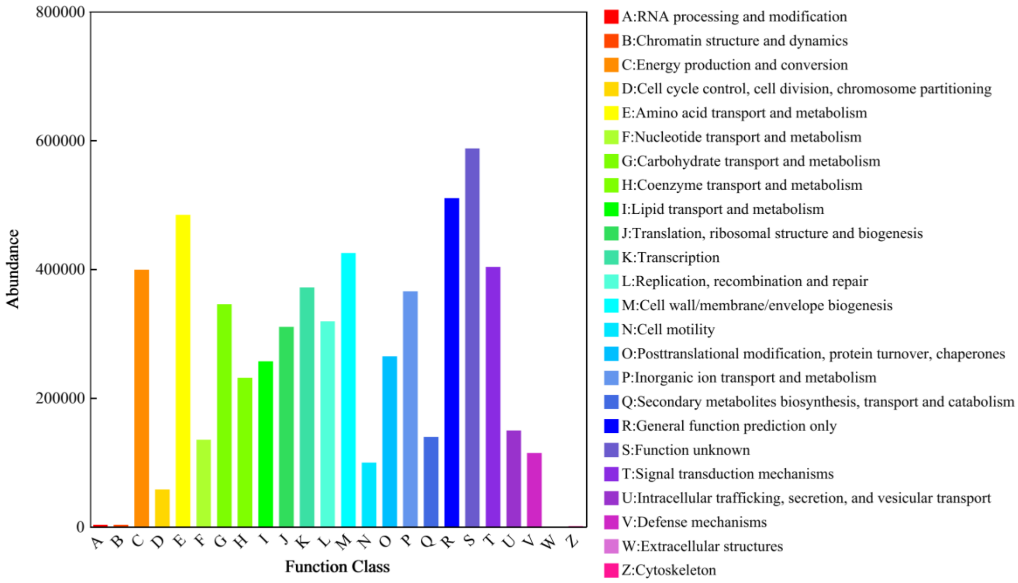
800000
600000
400000
200000
0
Abundance
A B C D E F G H I J K L M N O P Q R S T U V W Z
Function Class
A:RNA processing and modification
B:Chromatin structure and dynamics
C:Energy production and conversion
D:Cell cycle control, cell division, chromosome partitioning
E:Amino acid transport and metabolism
F:Nucleotide transport and metabolism
G:Carbohydrate transport and metabolism
H:Coenzyme transport and metabolism
I:Lipid transport and metabolism
J:Translation, ribosomal structure and biogenesis
K:Transcription
L:Replication, recombination and repair
M:Cell wall/membrane/envelope biogenesis
N:Cell motility
O:Posttranslational modification, protein turnover, chaperones
P:Inorganic ion transport and metabolism
Q:Secondary metabolites biosynthesis, transport and catabolism
R:General function prediction only
S:Function unknown
T:Signal transduction mechanisms
U:Intracellular trafficking, secretion, and vesicular transport
V:Defense mechanisms
W:Extracellular structures
Z:Cytoskeleton

(c)

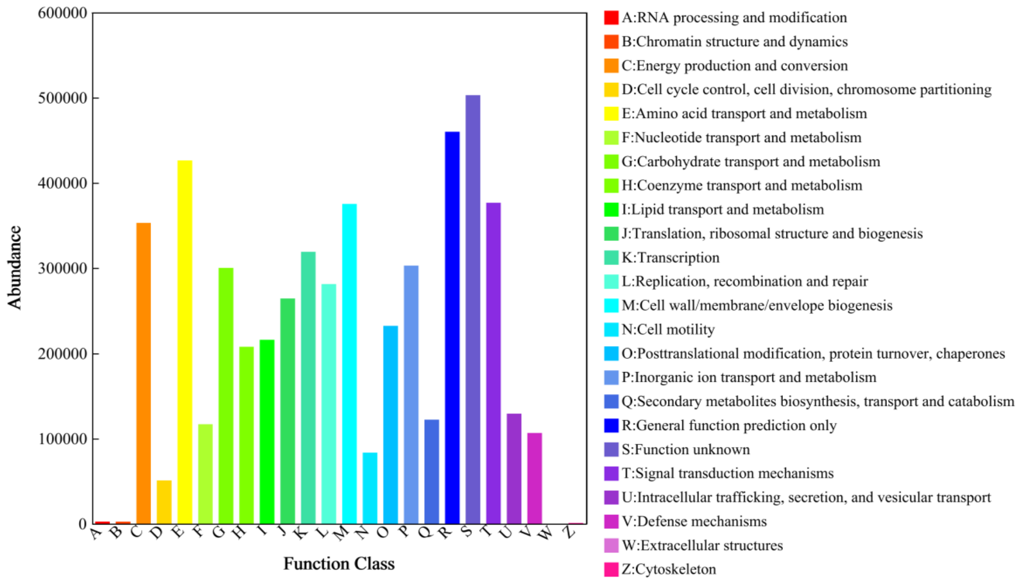
600000
500000
400000
300000
200000
100000
0
Abundance
A B C D E F G H I J K L M N O P Q R S T U V W Z
Function Class
A:RNA processing and modification
B:Chromatin structure and dynamics
C:Energy production and conversion
D:Cell cycle control, cell division, chromosome partitioning
E:Amino acid transport and metabolism
F:Nucleotide transport and metabolism
G:Carbohydrate transport and metabolism
H:Coenzyme transport and metabolism
I:Lipid transport and metabolism
J:Translation, ribosomal structure and biogenesis
K:Transcription
L:Replication, recombination and repair
M:Cell wall/membrane/envelope biogenesis
N:Cell motility
O:Posttranslational modification, protein turnover, chaperones
P:Inorganic ion transport and metabolism
Q:Secondary metabolites biosynthesis, transport and catabolism
R:General function prediction only
S:Function unknown
T:Signal transduction mechanisms
U:Intracellular trafficking, secretion, and vesicular transport
V:Defense mechanisms
W:Extracellular structures
Z:Cytoskeleton

(d)

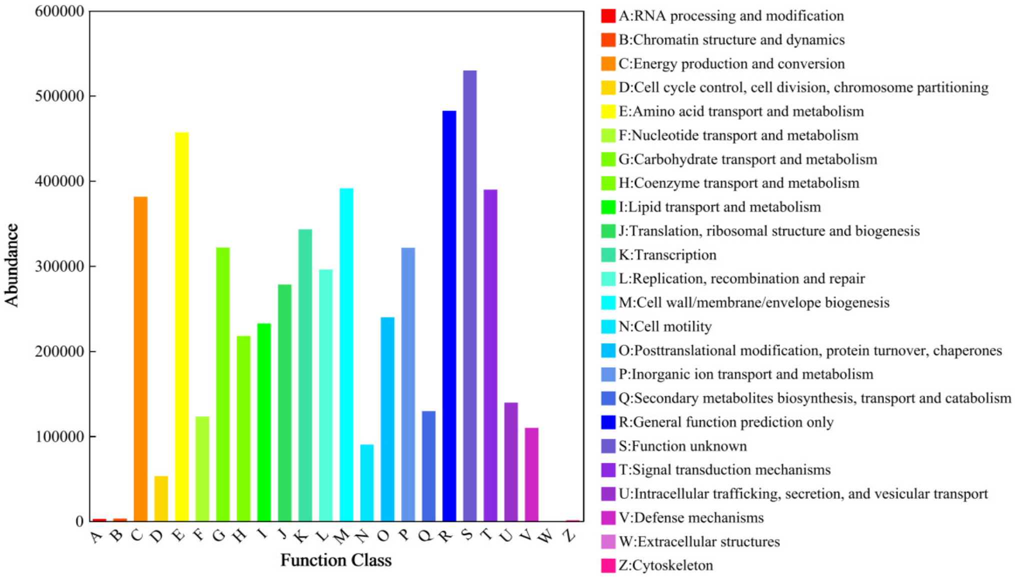

(e)

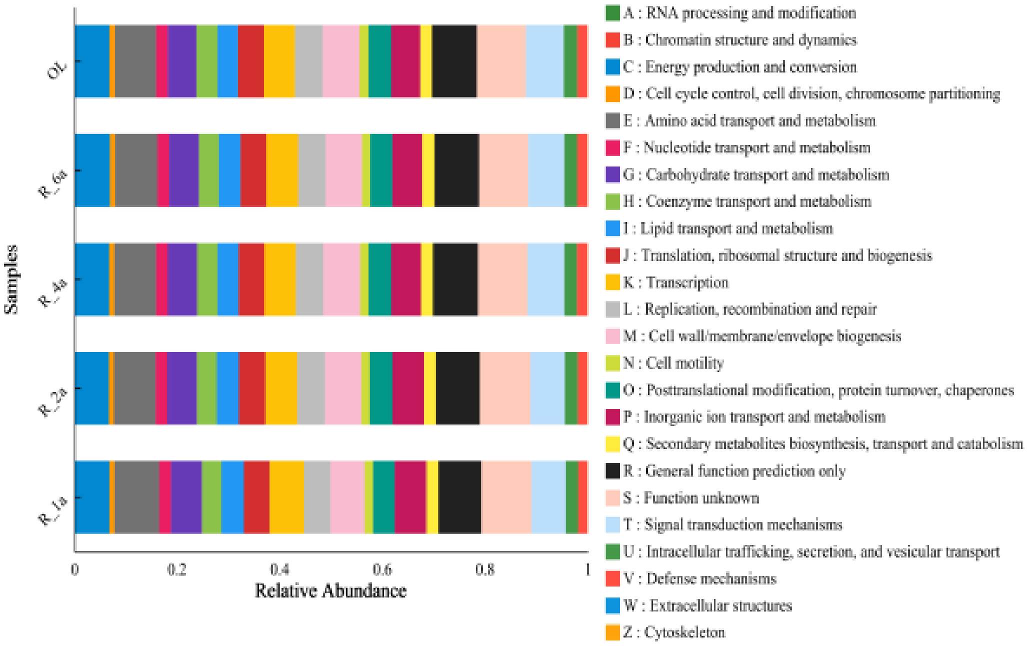

(f)

Figure S6. Functional profiles of the bacterial communities obtained using PICRUStl analysis. (a)OL(b)R_1a(c)R_2a(d)R_4a(e)R_6a(f)GOG function classification.

Table S1. The VIF values of the selected environmental factors after screening.

	pH	EC	CEC	SBD	STP	SCP	SWC	TN	SOM	HN	AP	AK
VIF	1.06	1.39	1.43	1.04	4.79	4.76	1.50	1.23	1.67	1.57	3.99	4.83

Table S2. RDA -related data RDA.

	RDA1	RDA2	r^2	p_values
AK	0.5158	-0.8567	0.9936	0.0083
TN	-0.8556	-0.5176	0.9899	0.0333
SOM	-0.8409	0.5411	0.882	0.0417
EC	0.9712	-0.2383	0.8985	0.1083
DM	0.9186	-0.3951	0.8092	0.1333
AP	0.4558	-0.8901	0.7864	0.175
HN	-0.9508	0.3099	0.5259	0.4167
SPG	0.9277	-0.3732	0.6421	0.4417
SWC	-0.8477	0.5304	0.2216	0.5417
SBD	0.7398	-0.6728	0.2705	0.55
CEC	-0.8962	-0.4437	0.3589	0.5583
STP	-0.6919	0.722	0.2014	0.7167
pH	0.2375	-0.9714	0.0956	0.9167
